# Intracellular evaluation of protein droplet-forming capability using self-assembling peptide tags†

**DOI:** 10.1039/d5sc00871a

**Published:** 2025-07-11

**Authors:** Takayuki Miki, Masahiro Hashimoto, Masatoshi Shimizu, Hiroki Takahashi, Hisakazu Mihara

**Affiliations:** a School of Life Science and Technology, Tokyo Institute of Technology 4259 Nagatsuta-cho, Midori-ku Yokohama Kanagawa 226-8501 Japan; b Department of Chemistry and Biotechnology, School of Engineering, The University of Tokyo 7-3-1 Hongo Bunkyo-ku Tokyo 113-0033 Japan miki@macro.t.u-tokyo.ac.jp

## Abstract

Protein droplet formation is a crucial process involved in transient cellular responses and pathogenic protein aggregations. Conventionally, the droplet-forming capability of target proteins has been evaluated through *in vitro* reconstitution studies, where purified proteins are dissolved in buffer solutions. However, such droplets are highly sensitive to environmental factors, including temperature, ionic strength, and molecular crowding. Therefore, *in situ* evaluation within living cells is highly desirable. Additionally, since droplet formation is typically initiated by nucleation involving dynamic protein oligomerization, simply expressing proteins in cells often fails to induce droplet formation, making intracellular evaluation challenging. In this study, we present an intracellular droplet-forming assay based on our peptide tag technique. This system employs short self-assembling YK peptide tags (7–15 residues), genetically fused to target proteins, to artificially induce oligomerization. Using this approach, we discover that the co-chaperone Hsp70/Hsp90 organizing protein possesses droplet-forming capability and identify the essential region required for its droplet formation.

## Abstract 2

Fusion with a self-assembling peptide provides a simple readout for identifying droplet-prone proteins in a living cellular context.

## Graphical abstract

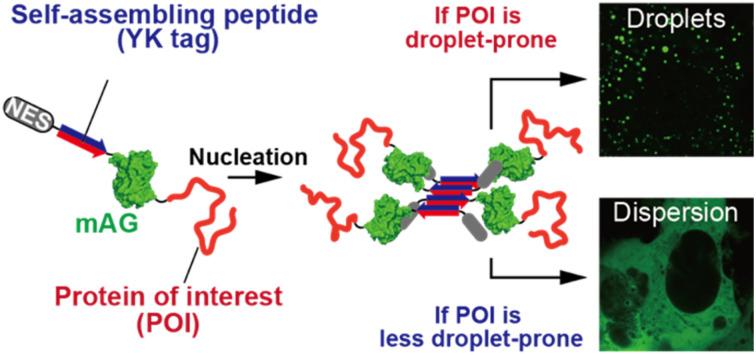

## Introduction

Protein droplets are ubiquitous within cells.^1,2^ Most of the droplet-forming proteins possess intrinsically disordered regions (IDRs).^3^ The entanglement of IDRs and promiscuous interactions, including electrostatic, π–cation, and π–π interactions, are thought to contribute to the assemblies.^4^ Extensive research on droplet-forming proteins revealed that over 100 proteins within the human proteome spontaneously undergo phase separation and have been categorized as droplet-driving proteins in databases.^5,6^ Moreover, sequence-based prediction estimates that approximately 40% of proteins in the human proteome are droplet-driving proteins.^7^ A large number of the predicted droplet-forming proteins and domains, however, have not yet been experimentally verified. The standard approach for studying droplet formation has relied on *in vitro* reconstitution using purified proteins in buffer solutions. However, phase separation is highly sensitive to solvent conditions, such as salt concentration,^8^ temperature,^9^ and molecular crowding,^10–12^ and the outcomes are dependent on the experimental conditions. Therefore, in addition to *in vitro* studies, it is critically essential to assess whether proteins of interest can undergo phase separation within the cellular context.

Oligomerization of folded domains or motifs often triggers phase separation. For instance, a droplet formation of galectin-3, which harbors an intrinsically disordered N-terminal domain, is driven by glycan-mediated oligomerization.^13^ Similarly, homo-oligomerization of the TDP-43 N-terminal domain lowers the phase diagram boundary.^14^ Furthermore, the Brangwynne group demonstrated that light-activated oligomerization using the Cry2 protein could drive phase separation of IDRs, including the fused in sarcoma (FUS) low-complexity (LC) domain, a representative droplet-driving domain.^15^ Using the optogenetic tool Corelets, they successfully mapped phase diagrams within intracellular conditions.^16^ Moreover, they showed that the classical nucleation theory can be applied to interpreting droplet formation observed in living cells.^17^ The optogenetic approach has also been applied to identify critical regions within a protein of interest for driving droplet formation in a cellular context.^10^

**Fig. 1:**
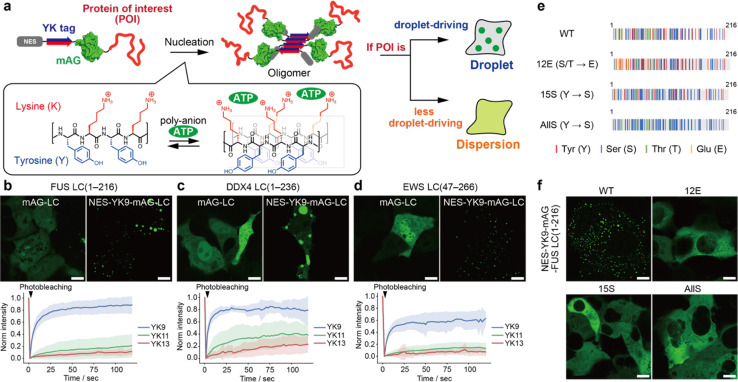
YK fusion-induced droplet formation of low-complexity (LC) domains. (a) Schematic illustration of the intracellular droplet-forming assay of a protein of interest. (b–d) Fluorescence images of the NES-YK9-mAG-fused LCs. The scale bars mean 10 μm. The fluorescence recovery after photobleaching (FRAP) results are shown in the lower panel. The line plots represent the mean values with shaded error bands (standard deviation [S.D.], *n* = 9 cells). (e) FUS LC domain (1–216) mutants (red: tyrosine; blue: serine; green: threonine; yellow: glutamate). (f) Fluorescence images of NES-YK9-mAG-FUS LC mutants (12E, 15S and AllS). The mutants with low droplet-forming capability were dispersed in the cytoplasm. The scale bars mean 10 μm.

In this study, given this information, we aim to devise an *in situ* evaluation method for the droplet-forming capability of proteins in living cells by inducing artificial oligomerization through short self-assembling peptide tags (Fig. 1a). In this system, the protein of interest (POI) is genetically fused with a self-assembling peptide tag containing 7–15 residues, a short size similar to that of commonly used epitope tags. Subsequently, droplet formation is assessed in living cells. If the POI possesses a high droplet-forming potential, the fusion forms fluidic liquid droplets. Conversely, proteins with low droplet-forming potential remain dispersed. For the self-assembling peptide tag, we use a *de novo* YK peptide that consists of alternating repeats of hydrophobic Tyr and cationic Lys residues, which we have recently reported.^18^ The YK peptides interact with adenosine triphosphate (ATP), an abundant polyanion species within cells, to form reversible fibrous structures. The number of YK repeats can modulate the self-association of the fused protein.

As a proof-of-concept, we demonstrate that the fusion of a short YK9 peptide induces intracellular droplet formation of the FUS LC domain, whereas negative control mutants fail to undergo phase separation. Using the YK-based intracellular evaluation, we discover that the co-chaperone Hsp70/Hsp90 organizing protein (HOP) has droplet-forming capability. This finding is corroborated by our *in vitro* test. Moreover, this method allows us to identify the self-assembling core motif within the HOP protein. Additionally, we observe that the artificially formed HOP droplets recruit endogenous Hsp70 and Hsp90, and their dynamic liquid-like nature is disrupted by adding Hsp inhibitors. These findings highlight the versatility of the YK peptide tag technique as a powerful tool for assessing droplet-forming proteins within cells.

## Results & discussion

### Intracellular evaluation of the droplet-forming capability of proteins by YK tagging

In our previous study, we observed that the fusion of the YK tag with weakly dimeric superfolder green fluorescent protein (sfGFP),^19^ along with the nuclear export signal (NES), resulted in droplet formation in the cytosolic space.^18^ In contrast, NES-YK fusion with monomeric fluorescent proteins, such as monomeric Azami-Green (mAG)^20^ and mCherry, did not lead to droplet formation. Despite their incapability of droplet formation, these YK-fusions formed oligomers through YK peptide self-assembly. NES-YK13-mAG exhibited slower diffusion in fluorescence correlation spectroscopy than mAG without YK tags (Fig. S1†). The reduction in fluorescence anisotropy associated with homo-FRET among clusters also supports their oligomerization.^18^ The droplet formation is highly dependent on the self-association of the fused proteins. This finding inspired us to develop a tool for assessing the droplet-forming capability of proteins in living cells. We hypothesized that proteins with strong droplet-forming capability would exhibit liquid-like droplets upon YK fusion, whereas those with weaker capability would not (Fig. 1a).

For the proof-of-principle experiment, we first employed the FUS LC domain,^21^ 1–216 amino acids, an extensively studied droplet-forming domain, as a model (Fig. 1b). We fused FUS LC with the NES-YK tag and mAG for visualization. As noted above, NES-YK13-mAG forms oligomers but does not produce droplets. When NES-YK9-mAG-FUS LC was expressed in COS-7 cells, droplets were observed inside the cells (Fig. 1b, upper right). These droplets exhibited a dynamic property, as shown by fluorescence recovery after photobleaching (FRAP) results, with a recovery half-time *t*_1/2_ of 5.8 ± 1.7 s (Fig. 1b, lower and Table S1†). In contrast, fusion with YK11 or YK13 resulted in aggregates with little mobility (Fig. 1b and S2†). Notably, mAG-FUS LC without tags did not form droplets within cells (Fig. 1b, upper left), indicating that phase separation of FUS LC is induced by YK tag fusion. This is consistent with reports showing that expression of the LC domain does not lead to droplet formation.^15^ Moreover, the fusion of a negative control peptide YK9(K4Y/Y5K), with the central residues (K4 and Y5) swapped, failed to induce droplets, highlighting the importance of the intact YK repeat (Fig. S3†).

To investigate the contribution of the YK tag to droplet formation in more detail, we quantified the intracellular protein concentration. The median concentration of control protein, mAG-FUS LC (without the YK tag), was 51 μM (IQR: 38–91 μM) (Fig. S4 and Table S2†). In contrast, NES-YK9-, NES-YK11-, and NES-YK13-mAG-FUS LC showed a two-phase distribution with 22 μM (IQR: 8–97 μM), 9 μM (IQR: 4–15 μM), and 4 μM (IQR: 2–9 μM) in the dilute phase. The reduction in concentration of dispersed FUS LC upon YK tag fusion indicates that the YK tag lowers the critical concentration required for droplet formation. Accordingly, the partition coefficient (the ratio of protein concentration in the dense phase to that in the dilute phase) also increased with YK tag length (Fig. S4†). Contrary to expectations, the protein concentration within the dense phase decreased in a chain length-dependent manner. We attribute this to the possibility that longer YK tags promote protein denaturation, leading to the formation of less-fluorescent aggregates. Consistent with this, longer YK tags resulted in reduced overall expression levels (as discussed later) and induced assemblies lacking fluidity, as confirmed by FRAP analysis. We also attempted to examine these trends in a test tube, but the YK fusion caused FUS LC to aggregate in buffer, preventing further evaluation (Fig. S5†). Similarly, other LC domains were examined: DDX4 LC (1–236 amino acids)^9^ and EWS LC (47–266 amino acids)^22^ showed high fluidity in the case of YK9 fusion, whereas YK11 or YK13 fusions markedly reduced their fluidities (Fig. 1c, d, S2 and Table S1†). In all cases, YK9–13 tagging resulted in a decrease in the fluorescence anisotropy, indicating that the fused mAGs were in close proximity (Fig. S6†). At the same time, we noticed that the band intensity in western blotting decreased as the YK tag length increased (Fig. S7†). Since these trends were not observed in the case of sfGFP fusion,^18^ we speculate that these decreased expression levels may be associated with denaturation or aggregate formation.

We further investigated FUS LC mutants with low droplet-forming potential as negative controls. Phosphorylation in the FUS LC domain prevented the droplet formation, and the phosphomimetic FUS LC(12E) mutant, in which Ser/Thr is replaced with Glu, is known to fail to form droplets in physiological conditions^23^ (Fig. 1e). Thus, we tested FUS LC(12E). The result showed that the mutation of S/T-to-E drastically diminished droplet formation, even when YK9–13 peptides were fused (Fig. 1f and S8†). In addition, we tested two mutants, namely, FUS LC(15S) and FUS LC(AllS), with 15 or all 27 Tyr residues substituted for Ser, respectively, which are reported to have lost the capability of hydrogel formation *in vitro* and barely associate with stress granules in cells.^21^ These Y-to-S mutants with YK9–13 tags resulted in discernible impairments in droplet formation (Fig. 1f and S8†). Compared with untagged mAGs, these mutants exhibit low fluorescence anisotropy, suggesting that the FUS LC mutants form oligomers but not droplets (Fig. S6†). Thus, YK tags can serve as a tool to assess whether the protein has a high potential to undergo droplet formation.

### YK-tagging assay reveals a droplet-forming capability of HOP

We applied our YK-based droplet-forming assay to chaperones and co-chaperones. The liquid-to-solid transition underlies pathogenic amyloid formation in various neuro-degenerative diseases.^24–26^ For example, a patient-derived mutant FUS(G156E) initially forms liquid-like droplets, but upon aging, these droplets lose their ability to fuse and transition into fibers.^25^ Molecular chaperones have been discussed for their role in maintaining the liquid-like state of droplets and preventing phase transitions into amyloid aggregation. Proteome analyses have identified various molecular chaperones and co-chaperones in stress granules and P-bodies.^27^ Heat shock protein 70 (Hsp70) is recruited to aberrant stress granules containing misfolded proteins.^28^ Experiments *in vitro* have revealed that molecular chaperones, *e.g.*, Hsp27, Hdj1 (class II Hsp40 protein), and Hsp70, prevent the liquid-to-solid transition of FUS protein^29–31^ and that HspB1 suppresses the aggregation of TDP-43.^32^ Beyond their role in maintaining droplets, intriguingly, Hdj1 and Hsp70 themselves drive phase separation.^30,31^ We also confirmed the droplet-forming capability of Hdj1 in living cells with the YK9 fusion tag (Fig. S9†).

**Fig. 2:**
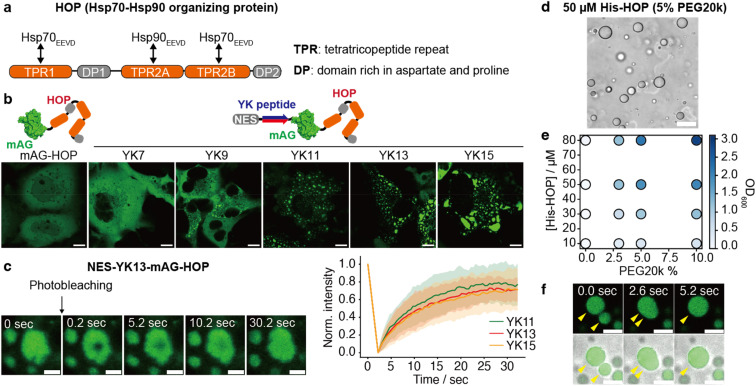
Cell-based droplet-forming assays for the HOP co-chaperone. (a) Domain organization of the HOP. (b) Fluorescence images of NES-YK-mAG-fused HOP in COS-7 cells. The scale bars mean 20 μm. (c) FRAP of NES-YK-mAG-HOP droplets. The left panel shows fluorescence time-lapse images of NES-YK13-mAG-HOP in the FRAP experiment. The right panel shows fluorescence recovery. The line plots represent the mean values with shaded error bands (S.D., *n* = 15 cells). (d) Bright-field image of His-HOP phase separation in a 5% PEG20k buffer. The scale bars mean 20 μm. (e) Turbidity (optical density at 600 nm) measurements across different PEG20k and His-HOP concentrations. The mean values are shown as a gradient color (*n* = 3 independent experiments). (f) Fusion of His-HOP droplets in a test tube. The scale bars mean 10 μm.

Given the important role of chaperones in maintaining liquid droplets, we set out to explore other droplet-forming proteins among chaperone-associated proteins. In general, chaperons do not exhibit a strong tendency to form liquid droplets. According to the liquid–liquid phase separation (LLPS) predictor PASP,^33^ only 25 out of 70 proteins (35.7%) annotated with the Gene Ontology term GO:0044183 (protein folding chaperone) are predicted to form droplets—a value comparable to that of the entire human proteome (36.9%). In this study, we focused on the co-chaperone HOP, Hsp70-Hsp90 organizing protein, whose droplet-forming potential had not been previously investigated. HOP consists of three tetratricopeptide repeat (TPR) domains (TPR1, TPR2A, and TPR2B) and two Asp-Pro-rich (DP) domains (Fig. 2a). TPR1/TPR2B and TPR2A bind for the C-terminal peptides (EEVD motif) of Hsp70 and Hsp90, respectively.^34,35^ HOP has been identified in proteome analyses of stress granules and P granules, and is categorized as Tier 1 protein in the RNA granule database^36^ (Fig. S10†). HOP has also been prominently identified in proteome analyses of purinosomes^37,38^—liquid-like condensates implicated in *de novo* purine biosynthesis—but its droplet-forming capability has been untested. Therefore, we employed our system with HOP. As a result, we found that HOP tagged with NES-YK11-, NES-YK13-, or NES-YK15-mAG formed droplets in cells (Fig. 2b and S11†), whereas the untagged mAG-HOP was dispersed. For the YK9 fusion, only a small subset of cells with high expression levels exhibited detectable droplets. From fluorescence intensity, we estimated that YK9 fusion was expressed at a median cytosolic concentration of 77 μM (IQR: 30–134 μM) (Fig. S12 and Table S3†). In contrast, fusions with YK11, YK13, and YK15 resulted in lower concentrations in the dilute phase with median values of 24 μM (IQR: 8–51 μM), 10 μM (IQR: 5–28 μM), and 27 μM (IQR: 10–42 μM), respectively. In the case of HOP, fusion with YK11 or longer was sufficient to reduce the critical concentration for phase separation, thereby enabling droplet formation. NES-YK13-mAG-HOP showed a lower partition coefficient (6.2; IQR: 4.7–7.9) compared to NES-YK9-mAG-FUS LC (14; IQR: 10–22), reflecting its weaker self-assembly capability than that of FUS LC. This difference may explain why a longer YK tag (YK11 or more) is necessary to induce droplet formation in HOP. Consistently, the YK repeat sequence proved to be critical, as NES-YK13(K6Y/Y7K)-mAG-HOP failed to form droplets (Fig. S13†). FRAP analysis confirmed the liquid-like dynamics of the condensates (Fig. 2c and Table S4†). Intriguingly, there are no significant differences in the dynamics among YK11, YK13, and YK15 fusions, contrasting with trends observed in other LC domains. Treatment with 1,6-hexanediol, which is widely used to disrupt weak hydrophobic interactions, did not dissolve HOP droplets (Fig. S14†). Sequence analysis revealed that HOP is rich in charged residues, ranking it in the top 4% of the human proteome. This suggests electrostatic, rather than hydrophobic, interactions drive its droplet formation. Collectively, our data identify HOP as a droplet-forming protein.

For the validation experiment, we purified His-tagged HOP without YK tags and evaluated its droplet formation *in vitro*. In test tubes, His-HOP formed highly spherical structures (roundness: 0.98 ± 0.01) in a PEG20k-containing solution (Fig. 2d, S15, and Movie S1†). The phase diagram based on turbidity tests showed that His-HOP undergoes phase separation at concentrations over 30 μM in the presence of PEG20k at concentrations above 3% (Fig. 2e). For detailed investigations, we chemically modified His-HOP with an Oregon Green fluorophore (Fig. S15†). FRAP experiments showed that the droplet dynamics were too fast to track the fluorescence recovery precisely (Fig. S16†). From time-lapse images, the fusion events between distinct droplets were readily observed (Fig. 2f). Thus, we experimentally demonstrated that the HOP could form droplets.

### Identification of the essential region in HOP for its droplet formation

**Fig. 3:**
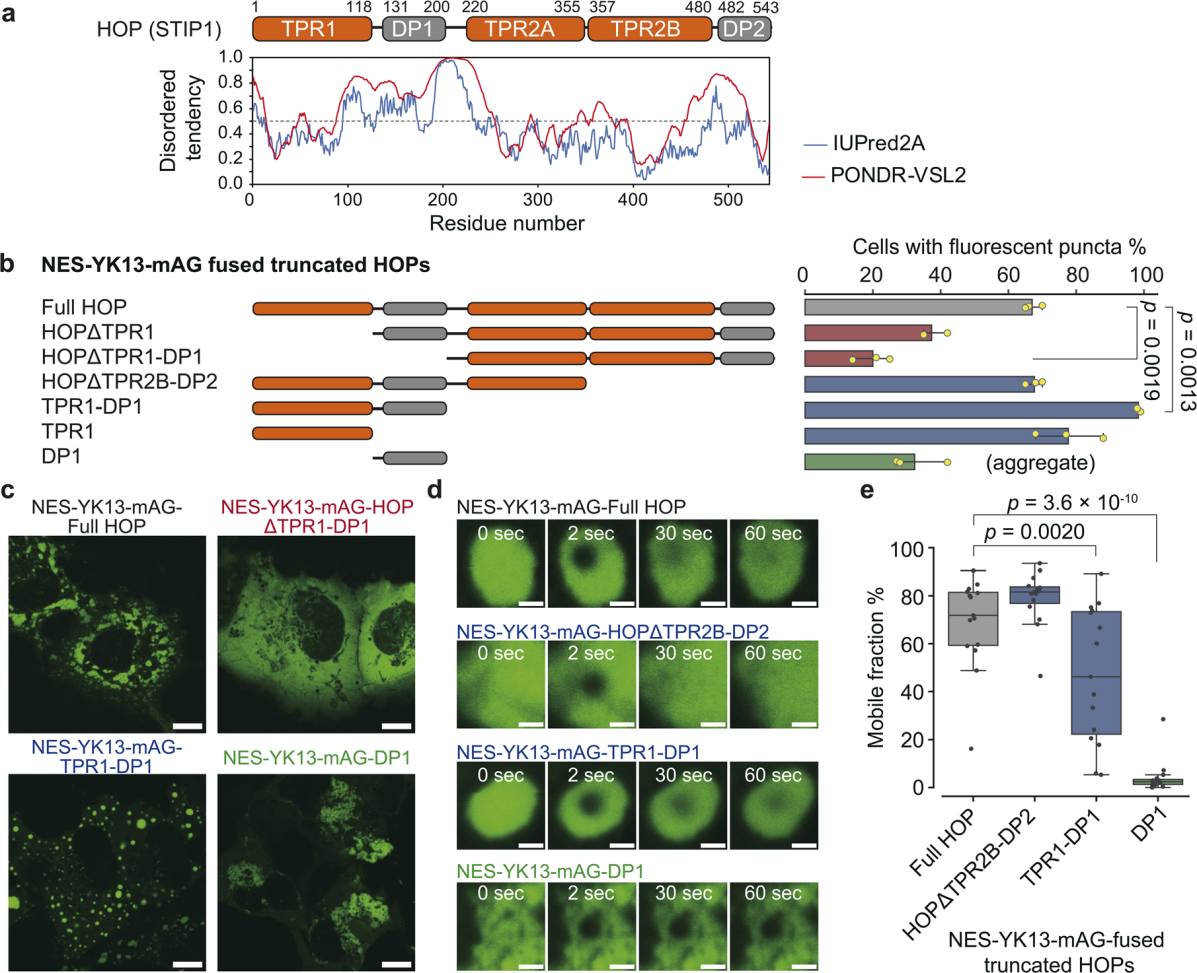
Identification of the essential region for HOP droplet formation. (a) Domain organization of HOP with IUPred2A prediction results. (b) Frequency of droplet formation among NES-YK13-mAG-fused truncated HOPs. The left panel shows truncated HOP constructs used in this assay. The corresponding results are presented in the right panel as mean values ±S.D. (*n* = 3, independent samples). (c) Fluorescence images of COS-7 cells expressing NES-YK13-mAG-fused truncated HOPs. The scale bars mean 10 μm. (d) FRAP of droplets formed by YK13-tagged truncated HOPs. Time-lapse FRAP images are shown in the left panel. The scale bars mean 1 μm. The fluorescence recoveries after bleaching are shown in the right panel. The data points are presented as the mean values with shaded error bands representing the S.D. (*n* = 15 cells). (e) Mobile fractions obtained from FRAP analysis. The box plots are presented as follows: central line, median; box limits, Q1 and Q3; whiskers, 1.5× interquartile range; and points, outliers. (*n* = 15 cells). Statistical comparisons between the two groups were performed using unpaired two-tailed Student's *t*-tests.

TPR1-DP1 domains are predicted to contain a disordered region by IUPred2A, a sequence-based prediction method^39^ (Fig. 3a). The TANGO algorithm^40^ predicts an aggregation-prone sequence in the DP1 domain (Fig. S17†). Given these predictions, we hypothesized that the TPR1-DP1 segment could be a self-assembling moiety for droplet formation.

We fused NES-YK13-mAG to truncated HOP proteins and assessed the frequency of droplet formation (Fig. 3b, c, and S18†). Truncation of the N-terminal domains (TPR1 or TPR1-DP1), as in HOPΔTPR1 and HOPΔTPR1-DP1 fusions, reduced the proportion of cells exhibiting fluorescent droplets. In contrast, nearly all cells expressing TPR1-DP1 fusion exhibited fluorescent spherical puncta. The median roundness values for the TPR1 and TPR1-DP1 fusions were 0.78 (IQR: 0.67–0.87) and 0.81 (IQR: 0.71–0.89), respectively, which were higher than that of the full-length HOP fusion (0.71; IQR: 0.57–0.82) (Fig. S19 and Table S5†). DP1 fusion resulted in dynamically arrested large aggregates, as evidenced by the lack of fluorescence recovery in FRAP analysis (Fig. 3d, e and Table S6†), and exhibited a low roundness value (0.57; IQR: 0.45–0.70), consistent with TANGO prediction. These findings underscore the contribution of TPR1-DP1, the N-terminal region of HOP, to droplet formation. This finding is intriguing since the biochemical role of TPR1-DP1 remains poorly understood, while the C-terminal segment of HOP, TPR2A-TPR2B-DP2, is sufficient for assisting in the full activation of a client protein (glucocorticoid receptor).^41^ Of note, *Drosophila melanogaster* and *Caenorhabditis elegans* lack DP1 and TPR1-DP1 domains, respectively.^42,43^ Recently, the complex structure of HOP with Hsp70, Hsp90, and a client protein has been determined,^34^ where only the TPR2A-TPR2B-DP2 segments have been observed despite the whole sequence of recombinant HOP being used (Fig. S17†).

### Maintenance of HOP-based droplets by chaperones

**Fig. 4:**
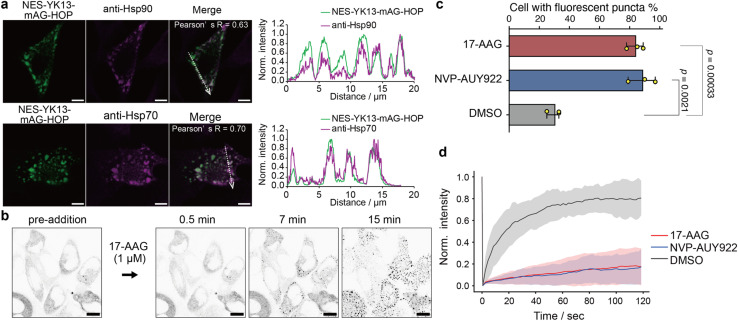
Contribution of the molecular chaperones Hsp70 and Hsp90 to the maintenance of HOP droplets. (a) Colocalization analysis of NES-YK13-mAG-HOP with endogenous Hsp90 (top) and Hsp70 (bottom) in COS-7 cells. Pearson's correlation coefficients (*R* values) are indicated. The right panels show normalized fluorescence intensity line-scan profiles along the dotted arrows. The scale bars mean 5 μm. (b) Time-lapse images of NES-YK13-mAG-HOP-expressing cells treated with the Hsp90 inhibitor 17-AAG. Inverted grayscale images are shown to highlight fluorescence changes. The scale bars mean 10 μm. (c) Quantification of the percentage of transfected cells displaying fluorescent puncta upon treatment with 17-AAG, NVP-AUY922, or DMSO. Data represent the mean ± S.D. from three biologically independent experiments. Statistical significance was determined using unpaired two-tailed Student's *t*-tests. (d) FRAP analysis following inhibitor treatment. The line plots represent the mean values with shaded error bands (S.D., *n* = 15 cells).

Next, we investigated whether Hsp70 and Hsp90 were recruited to the HOP droplets formed by YK tagging in HeLa cells. Immunofluorescence images showed clear colocalization of endogenous Hsp70 and Hsp90 with the HOP-based droplets (Fig. 4a and S20†). We wondered whether these chaperones would regulate the formation of HOP droplets and their dynamic features. We treated HeLa cells expressing NES-YK13-mAG-HOP with 17-AAG or NVP-AUY922, Hsp90 inhibitors. Upon the addition of Hsp90 inhibitors, the number of fluorescent puncta dramatically enhanced in approximately 30 minutes (Fig. 4b, S21, and Movie S2†). The droplet formation frequency dramatically increased from 30 ± 5% (DMSO control) to 84 ± 6% and 89 ± 9% after 17-AAG or NVP-AUY922 treatment, respectively (Fig. 4c and S22†), although the frequency under normal conditions in HeLa cells is lower than that in COS-7 cells. It is worth noting that these bodies appeared to be dynamically arrested (Fig. 4d and Table S7†). Similarly, adding an Hsp70 inhibitor (VER-155008) increased the number of fluorescent puncta and emerged aggregates (Fig. S23†). In the negative control cells expressing NES-YK13-sfGFP or NES-YK9-mAG-FUS LC, we could not find any significant influence of these Hsp inhibitors on the droplet formation or dynamic properties (Fig. S24 and S25†). These results indicate that Hsp70 and Hsp90 are maintaining the dynamic properties of the HOP droplets by preventing their aggregation.

## Conclusions

In this study, we developed an intracellular assessment for the droplet-forming capability of protein by leveraging our self-assembling peptide tag technique. As a proof-of-concept, we confirmed that YK peptide fusion induced droplet formation of model LC domains. In contrast, FUS mutants with low droplet-forming potential remained dispersed in cells even after YK peptide fusion. Applying this method to HOP, a protein whose droplet-forming capability had not been experimentally tested, we discovered that YK-fused HOP formed droplets. Furthermore, domain truncation experiments identified the N-terminal TPR1-DP1 domains as critical for phase separation.

The advantage of our method over optogenetic tools is its simplicity. Our method only requires the fusion of short peptides comprising 7–15 residues,^44–47^ similar in size to the widely used epitope tags. The genes for these peptides can be quickly introduced using general techniques such as insertion of annealed oligo DNA or inverse PCR, making it a widely applicable technology for most laboratories.^48^ Additionally, the small size of the tag is advantageous for tasks such as library construction.^49^ Since the droplets form spontaneously upon expression, this system offers the advantage of highly reproducible data acquisition without requiring specific experimental setups such as light irradiation. Moreover, because continuous illumination is unnecessary, it also allows for the observation of time-dependent changes over several days, such as aging processes. Indeed, the fluidity of NES-YK9-mAG-FUS LC droplets was found to depend on incubation time: on day 1 after transfection, fluorescence recovery in FRAP experiments was faster than on day 2 (Fig. S26†). One drawback of our system is that it is unsuitable for investigating the time course of droplet formation.

Considering that an estimated 40% of the human proteome could be droplet-forming proteins,^7^ our technique offers a convenient tool for analyzing droplet formation under physiological conditions through a bottom-up approach. Moreover, it is effective in identifying critical regions for droplet formation. Indeed, our study revealed that HOP had a high potential for droplet formation and that the N-terminal region, the physiological role of which had been previously unclear, was prone to phase separation. In conclusion, as demonstrated above, YK peptides represent a beneficial tool for intracellularly evaluating protein droplet-forming ability.

## Statistics & reproducibility

The sample size for biochemical studies was set at three. For cell image analysis, we obtained three to ten different images from various fields of view in a single biological experiment. We conducted three biologically independent experiments. Statistical comparisons between the two groups were performed using unpaired two-tailed Student's *t*-tests.

## Author contributions

T. M. designed the project. T. M., M. H., M. S., and H. T. constructed the expression plasmids. T. M. expressed and purified proteins and tested their droplet formation in a test tube. T. M., M. H., M. S., and H. T. perform cell experiments. According to the discussion with all authors, the manuscript was written by M. H., H. M., and T. M.

## Conflicts of interest

All authors declare no competing interests.

## Supplementary Material

SC-016-D5SC00871A-s001

SC-016-D5SC00871A-s002

SC-016-D5SC00871A-s003

## Data Availability

Data supporting the findings of this study are included in the article, along with ESI.†
