# Supplementary material for: Intracellular evaluation of protein droplet-forming capability using self-assembling peptide tags
Source: Chem Sci. 2025 Jul 11;16(31):14384–91. doi: 10.1039/d5sc00871a (PMC12247213; doi:10.1039/d5sc00871a)
Supplement: SC-016-D5SC00871A-s003 [file SC-016-D5SC00871A-s003.pdf]

Supplementary Information (SI) for Chemical Science.
This journal is © The Royal Society of Chemistry 2025

Supporting Information for

Intracellular evaluation of protein droplet-forming capability using self-assembling peptide tags

Takayuki Miki[1,2*], Masahiro Hashimoto[1‡], Masatoshi Shimizu[2‡], Hiroki Takahashi[1], Hisakazu Mihara[1]

1. School of Life Science and Technology, Tokyo Institute of Technology, 4259 Nagatsuta-cho, Midori-ku, Yokohama, Kanagawa 226-8501, Japan
2. Department of Chemistry and Biotechnology, School of Engineering, The University of Tokyo, 7-3-1 Hongo, Bunkyo-ku, Tokyo 113-0033, Japan.

Table of Contents

Material & Methods S3
Table S1 FRAP results of NES-YK-mAG-LCs S6
Table S2 Intracellular concentrations of FUS LC fused with different lengths of YK peptides S6
Table S3 Intracellular concentrations of HOP proteins fused with different lengths of YK peptides S6
Table S4 FRAP results of NES-YK-mAG-HOPs S6
Table S5 Roundness and size of fluorescent puncta formed by HOP and truncated HOP fusions ...S7
Table S6 FRAP results of NES-YK-mAG-domain deficient HOPs S7
Table S7 FRAP results of NES-YK-mAG-HOP following Hsp90 inhibitor treatments S7
Table S8 Sequence of proteins tested in this study S8
Table S9 Primer DNAs for cloning. S14
Figure S1 Live cell FCS analysis of mAG-HA and NES-YK13-mAG S15
Figure S2 FRAP analysis of NES-YK-mAG-LCs in COS-7 cells S16
Figure S3 Fluorescence images of COS-7 cells expressing NES-YK9(K4Y/Y5K)-mAG FUS LC S17
Figure S4 Quantification of intracellular mAG-FUS LC concentrations and partition coefficient in COS-7 cells S18
Figure S5 YK-fused FUS LC forms aggregates in vitro but remains soluble in mammalian cells S19
Figure S6 Fluorescence anisotropy of NES-YK-mAG-LCs in COS-7 cells S20
Figure S7 Western blotting of COS-7 cells expressing NES-YK-mAG-LC S21
Figure S8 Fluorescence images of COS-7 cells expressing NES-YK-mAG FUS LC mutants S22
Figure S9 Droplet formation of YK-tagged Hdj1 in living cells S23
Figure S10 HOP (STIP1) in RNA granule database S24

Figure S11 Fluorescence images of COS-7 cells expressing NES-YK-mAG-HOPs S24
Figure S12 Quantification of intracellular mAG-HOP concentrations in COS-7 cells S25
Figure S13 Fluorescence images of COS-7 cells expressing NES-YK13(K6Y/Y7K)-mAG HOP S26
Figure S14 Resistance of NES-YK13-mAG-HOP droplets to 1,6-hexanediol treatment S27
Figure S15 Droplet formation of His-HOP in test tubes S28
Figure S16 FRAP of His-HOP in test tubes S29
Figure S17 Prediction of aggregation tendency and structure of HOP S30
Figure S18 Fluorescence images of COS-7 cells expressing NES-YK13-mAG-fused truncated HOPs S31
Figure S19 Roundness and size analysis of fluorescent puncta formed by HOP and truncated HOP fusions in living cells S32
Figure S20 Colocalization of endogenous Hsp70 and Hsp90 with NES-YK13-mAG-HOP droplets S33
Figure S21 Hsp90 inhibitor NVP-AUY922 inducing NES-YK13-mAG-HOP aggregation S34
Figure S22 Increase in the frequency of cells exhibiting fluorescent puncta after Hsp90 inhibitor treatments S35
Figure S23 FRAP of NES-YK13-mAG-HOP droplets after Hsp70 inhibitor addition S36
Figure S24 Hsp70 or Hsp90 inhibitors have no significant effect on NES-YK13-sfGFP droplets S37
Figure S25 Hsp70 or Hsp90 inhibitors have no significant effect on NES-YK9-mAG-FUS LC droplets S38
Figure S26 FRAP results of NES-YK9-mAG-FUS LC droplet on different incubation days S38

MATERIALS AND METHODS

Design and construction of plasmids.

For the construction of plasmids encoding NES-YKn-mAG-fused proteins, the corresponding genes were cloned into a pCI-neo vector (Promega, E1841) by a standard subcloning method[1]. DNA oligos encoding NES-peptides were ordered (FASMAC), phosphorylated by T4 polynucleotide kinase (PNK, Takara), incubated at 70 °C for 20 min to inactivate PNK, incubated at 90 °C for 5 min to denature DNA, and cooled at −1 °C/min for DNA annealing. The gene encoding mAG or mCherry was inserted between the EcoRI–SalI site. The genes for the IDR were purchased from GeneArt Gene Synthesis (Thermo), and the gene for HOP was cloned from the Human Brain, Whole Marathon®-Ready cDNA (Clontech), as previously reported[2]. The genes for domain-truncated HOPs were prepared by PCR using Q5 polymerase (New England Biolabs). We inserted these genes between the SalI–NotI site. For the expression of HOP in bacterial cells, the HOP genes were cloned into pET vectors between the NheI–NotI site.

Mammalian cell culture and transfection.

HeLa and COS-7 cells were provided by RIKEN BioResource Research Center (BRC) through the National BioResource Project of the MEXT/AMED, Japan. These cells were cultured in DMEM (043-30085, Fujifilm) supplemented with 10% FBS (F7524, Sigma–Aldrich) and penicillin/streptomycin (Nacalai Tesque) under a humid 5% CO_2 atmosphere at 37 °C. Then, 1–2×10^4 cells/well were seeded on an EZVIEW 96-well culture plate (AGC) 1 day before transfection. In accordance with the manual, the COS-7 or HeLa cells were transfected with 100 ng of plasmid DNA using Lipofectamine® 3000 (Thermo Fisher Scientific) or X-tremeGENE HP (Roche). Two days after transfection, the medium was exchanged with DMEM (phenol red free, 044-32955, Fujifilm), and the cells were assessed.

Fluorescence observation and intracellular FRAP analysis.

The transfected cells were observed on a Zeiss LSM 780 confocal microscope equipped with a 63×/1.4 NA oil immersion objective using ZEN 2.3 (black edition) software (488-nm argon laser for mAG and sfGFP; 561-nm DPSS laser for mCherry; and 633-nm HeNe 633 laser for Alexa Fluor® 647) or Leica TCS SP8 confocal microscope equipped with a 63×/1.4NA oil immersion objective. For FRAP analysis, the circular regions of interest were bleached with 100% power of a 488 nm laser, followed by time-lapse observation. The fluorescence recovery curves were fitted in a single exponential mode to calculate the half-time and mobile fraction. For in vitro studies, protein solutions were placed on a 96-well EZVIEW culture plate (AGC) and similarly examined.

Image Analysis.

The roundness of fluorescent puncta was quantified using Fiji (ImageJ). Droplet regions were defined by Otsu's thresholding method and analyzed using the Particle Analyzer plugin, with size constraints set between 0.1 and 500 μm². Intracellular protein concentrations were estimated according to our previously established protocol[3]. A standard curve was generated from serial dilutions of recombinant mAG, enabling the conversion of fluorescence intensity to concentration values. Cell regions in each fluorescence image were segmented using percentile (0.5) thresholding. Areas exhibiting fluorescence intensities 50% higher than the average intensity of the cell region were defined as the dense phase, while the remaining area was designated as the dilute phase. Mean fluorescence intensities in each phase were then converted to protein concentrations using the standard curve.

Preparation of recombinant HOP and Oregon Green-modified HOP.

Using conventional subcloning techniques, we prepared pET28a vector encoding HOP. The transformed ECOS™ SONIC Escherichia coli Competent Cells [BL21(DE3) derived] (Nippon Gene) were incubated in an LB medium with kanamycin. After induction with 1 mM isopropyl β-d-1-thiogalactopyranoside (IPTG) for 5 hours at 30 °C, the cells were harvested by centrifugation (6,000 × g, 15 min, 4 °C). The cell pellets were suspended in 50 mM sodium phosphate and 300 mM NaCl buffer containing protease inhibitor cocktail set VII (Fujifilm) and homogenized by ultrasonication (UDS-200, Tomy) on ice. The soluble fraction obtained by centrifugation was applied to Talon resin as described for the YK-sfGFP purification. The eluate buffer was exchanged by ultrafiltration using an Amicon® Ultra filter (50 kDa cut-off). For fluorescence modification, 50 μM purified His-HOP solution was mixed with 500 μM Oregon Green™ 488 carboxylic acid, succinimidyl ester, 5-isomer (Invitrogen) for 5 hours at room temperature (approximately 20 °C) in 100 mM sodium phosphate buffer (pH 8.0). The HOP proteins were purified by ultrafiltration using an Amicon® Ultra filter (50 kDa cut-off), followed by buffer exchange using a Zeba™ spin desalting column (7 kDa cut-off, Thermo) into 20 mM sodium phosphate and 150 mM NaCl (pH 7.0) buffer. The protein concentration and Oregon Green modification yield were determined by UV-Vis absorption (280 nm and 495 nm).

Immunofluorescence images of HeLa cells expressing NES-YK13-sfGFP using anti-Hsp70 and anti-Hsp90 antibodies.

HeLa cells were transfected with plasmid DNA encoding NES-YK13-mAG-HOP. Two days post-transfection, the cells were fixed using 4% paraformaldehyde in phosphate buffer for 10 min at room temperature (20 °C), followed by permeabilization using 0.1% Triton X-100/PBS for 20 min at room temperature. Subsequently, after blocking with 10% normal goat serum (NGS)/PBS for 30 min, the samples were incubated overnight at 4 °C with either a rabbit polyclonal anti-Hsp70 antibody

(Proteintech, 10995-1-AP, 1:200 dilution) or a mouse monoclonal anti-Hsp90 antibody (StressMarq, SMC-107A, 1:200 dilution), both in 1% NGS/PBS solution. Following three PBS rinses, the samples were incubated with either a goat anti-rabbit IgG H&L (Alexa Fluor® 647) antibody (ab150079, 1:200 dilution) or a goat anti-mouse IgG H&L (Alexa Fluor® 647) antibody (ab150115, 1:200 dilution) in 1% NGS/PBS for 1 hour at room temperature.

Table S1 FRAP results of NES-YK-mAG-LCs

LC	FUS LC	FUS LC	FUS LC	DDX4 LC	DDX4 LC	DDX4 LC	EWS LC	EWS LC	EWS LC
YK tag	YK9	YK11	YK13	YK9	YK11	YK13	YK9	YK11	YK13
t half / sec	5.8 ± 1.7	36 ± 21	32 ± 17	3.5 ± 1.8	21 ± 10	36 ± 17	5.5 ± 1.4	21 ± 18	26 ± 22
Mobile fraction %	83 ± 12	20 ± 17	11 ± 5	80 ± 9	41 ± 19	24 ± 14	55 ± 16	15 ± 9	11 ± 8

Table S2 Intracellular concentrations of FUS LC fused with different lengths of YK peptides.

	mAG-FUS LC	NES-YK9-mAG-FUS LC	NES-YK11-mAG-FUS LC	NES-YK13-mAG-FUS LC
**Dilute phase**	51 µM (IQR: 38–91 µM)	22 µM (8–97 µM)	9 µM (IQR: 4–15 µM)	4 µM (2–9 µM)
**Dense phase**	–	480 µM (117–1,119 µM)	335 µM (163–701 µM)	223 µM (135–343 µM)
**Partition coefficient**	–	14 (10–22)	44 (23–69)	40 (27–83)

*Data are presented as median with interquartile range (IQR: Q1–Q3), as the distribution did not follow normality (Shapiro-Wilk test, $p < 0.05$).

Table S3 Intracellular concentrations of HOP proteins fused with different lengths of YK peptides.

	mAG-HOP	NES-YK9-mAG-HOP	NES-YK11-mAG-HOP	NES-YK13-mAG-HOP	NES-YK15-mAG-HOP
**Dilute phase**	51 µM (IQR: 14–89 µM)	77 µM (30–134 µM)	24 µM (8–51 µM)	10 µM (5–28 µM)	27 µM (10–42 µM)
**Dense phase**	–	–	91 µM (31–208 µM)	65 µM (35–143 µM)	104 µM (79–148 µM)
**Partition coefficient**	–	–	5.2 (3.5–6.6)	6.2 (4.7–7.9)	4.3 (1.5–5.9)

*Data are presented as median with interquartile range (IQR: Q1–Q3), as the distribution did not follow normality (Shapiro-Wilk test, $p < 0.05$).

Table S4 FRAP results of NES-YK-mAG-HOPs

	NES-YK11-mAG-HOP	NES-YK13-mAG-HOP	NES-YK15-mAG-HOP
t half / sec	7.8 ± 7.7	7.3 ± 5.2	8.0 ± 5.6
Mobile fraction %	86 ± 15	79 ± 14	77 ± 21

Table S5 Roundness and size of fluorescent puncta formed by HOP and truncated HOP fusions

	FULL	TPR1-DP1	TPR1	DP1
**Roundness**	0.71<br>(0.57–0.82)	0.81<br>(0.71–0.89)	0.78<br>(0.67–0.87)	0.57<br>(0.45–0.70)
**Area / µm^2**	0.54<br>(0.24–1.32)	0.40<br>(0.21–0.92)	0.23<br>(0.14–0.40)	0.33<br>(0.17–1.27)
n	3,270	2,026	6,667	512

Table S6 FRAP results of NES-YK-mAG-domain deficient HOPs

	HOP Full<br>1–543	HOPΔTPR2B-DP2<br>1–356	TRP1-DP1<br>1–200	DP1<br>119–200
**t half / sec**	12 ± 5	6.2 ± 2.8	22 ± 21	8.7 ± 12
**Mobile fraction %**	69 ± 19	79 ± 11	47 ± 28	4 ± 7

Table S7 FRAP results of NES-YK-mAG-HOP following Hsp90 inhibitor treatments

	DMSO	17-AAG	NVP-AUY922
**t half / sec**	15 ± 13	38 ± 34	37 ± 33
**Mobile fraction %**	82 ± 31	19 ± 22	19 ± 11

Table S8 Sequence of proteins tested in this study.

Protein	Sequence
NES-YK13-sfGFP	MDLQKKLEELELDEQQKKRLEFGGSGGYKYKYKYKYKYKYGGSVDMVSKGEELFTGVVPILVELDGDVNGHKFSVRGEGEGDATNGKLTLKFICTTGKLPVPWPTLVTTLTYGVQCFSRYPDHMKQHDFFKSAMPEGYVQERTISFKDDGTYKTRAEVKFEGDTLVNRIELKGIDFKEDGNILGHKLEYNFNSHNVYITADKQKNGIKANFKIRHNVEDGSVQLADHYQQNTPIGDGPVLLPDNHYLSTQSVLSKDPNEKRDHMVLLEFVTAAGITLGMDELYK
NES-YK13-mAG	MDLQKKLEELELDEQQKKRLEFGGSGGYKYKYKYKYKYKYGGSVDMVSVIKPEMKIKLCMRGTVNGHNFVIEGEGKGNPYEGTQILDLNVTEGAPLPFAYDILTTVFQYGNRAFTKYPADIQDYFKQTFPEGYHWERSMTYEDQGICTATSNISMRGDCFFYDIRFDGTNFPPNGPVMQKKTLKWEPSTEKMYVEDGVLKGDVNMRLLLEGGGHYRCDFKTTYKAKKEVRLPDAHKIDHRIEILKHDKDYNKVKLYENAVARYSMLPSQAK
NES-YK9-mAG-FUS(1–216)	MDLQKKLEELELDEQQKKRLGGSGGYKYKYKYKYGGSEFMVSVIKPEMKIKLCMRGTVNGHNFVIEGEGKGNPYEGTQILDLNVTEGAPLPFAYDILTTVFQYGNRAFTKYPADIQDYFKQTFPEGYHWERSMTYEDQGICTATSNISMRGDCFFYDIRFDGTNFPPNGPVMQKKTLKWEPSTEKMYVEDGVLKGDVNMRLLLEGGGHYRCDFKTTYKAKKEVRLPDAHKIDHRIEILKHDKDYNKVKLYENAVARYSMLPSQAKVDMASNDYTQQATQSYGAYPTQPGQGYSQQSSQPYGQQSYSGYSQSTDTSGYGQSSYSSYGQSQNTGYGTQSTPQGYGSTGGYGSSQSSQSSYGQQSSYPGYGQQPAPSSTSGSYGSSSQSSSYGQPQSGSYSQQPSYGGQQQSYGQQQSYNPPQGYGQQNQYNSSSGGGGGGGGGNYGQDQSSMSSGGGSGGGYGNQDQSGGGGSGGYGQQDRGGR
NES-YK9(K4Y/Y5K)-mAG-FUS(1–216)	MDLQKKLEELELDEQQKKRLGGSGGYKYYKKYKYGGSEFMVSVIKPEMKIKLCMRGTVNGHNFVIEGEGKGNPYEGTQILDLNVTEGAPLPFAYDILTTVFQYGNRAFTKYPADIQDYFKQTFPEGYHWERSMTYEDQGICTATSNISMRGDCFFYDIRFDGTNFPPNGPVMQKKTLKWEPSTEKMYVEDGVLKGDVNMRLLLEGGGHYRCDFKTTYKAKKEVRLPDAHKIDHRIEILKHDKDYNKVKLYENAVARYSMLPSQAKVDMASNDYTQQATQSYGAYPTQPGQGYSQQSSQPYGQQSYSGYSQSTDTSGYGQSSYSSYGQSQNTGY

	GTQSTPQGYGSTGGYGSSQSSQSSYGQQSSYPGYGQQPAPSST SGSYGSSSQSSSYGQPQSGSYSQQPSYGGQQQSYGQQQSYNP PQGYGQQNQYNSSSGGGGGGGGGNYGQDQSSMSSGGGSG GGYGNQDQSGGGGSGGYGQQDRGGR
NES-YK9-mAG-DDX4(1–236)	NES-YK9-mAG(same as the above sequence)-VDMGDEDWEAEINPHMSSYVPIFEKDRYSGENGDNFNRTPAS SSEMDDGPSRRDHFMKSGFASGRNFGNRDAGECNKRDNTST MGGFGVGKSFGNRGFSNSRFEDGDSSGFWRESSNDCEDNPTR NRGFSKRGGYRDGNNSEASGPYRRGGRGSFRGCRGGFGLGS PNNDLDPDECMQRTGGLFGSRRPVLSGTGNGDTSQSRSGSGS ERGGYKGLNEEVITGSGKNSWKSEAEGGES
NES-YK9-mAG-EWS(47–266)	NES-YK9-mAG-VDMPTDVSYTQAQTTATYGQTAYATSYGQPPTGYTTPTAPQA YSQPVQGYGTGAYDTTTATVTTTQASYAAQSAYGTQPAYPAY GQQPAATAPTRPQDGNKPTETSQPQSSTGGYNQPSLGYGQSN YSYPQVPGSYPMQPVTAPPSYPPTSYSSTQPTSYDQSSYSQQN TYGQPSSYGQQSSYGQQSSYGQQPPTSYPPQTGSYSQAPSQYS QQSSSYGQQSS
NES-YK9-mAG-FUS(1–216)12E	NES-YK9-mAG(same as the above sequence)-VDMASNDYEQQAEQSYGAYPEQPGQGYEQQSEQPYGQQSYS GYEQSTDTSGYGQSSYSSYGQEQNTGYGEQSTPQGYGSTGG YGSEQSEQSSYGQQSSYPGYGQQPAPSSTSGSYGSSEQSSSYG QPQSGSYEQQPSYGGQQQSYGQQQSYNPPQGYGQQNQYNSS SGGGGGGGGGNYGQDQSSMSSGGGSGGGYGNQDQSGGGG SGGYGQQDRGGR
NES-YK9-mAG-FUS(1–216)15S	NES-YK9-mAG(same as the above sequence)-VDMASNDYTQQATQSSGASPTQPGQGYSQQSSQPSGQQSSSG SSQSTDTSGYGQSSSSSSGQSQNTGYGTQSTPQGYGSTGGYGS SQSSQSSSGQQSSSPGSGQQPAPSSTSGSYGSSSQSSSYGQPQS GSSSQQPSYGGQQQSSGQQQSSNPPQGSGQQNQSNSSSGGGG GGGGGGNYGQDQSSMSSGGGSGGGYGNQDQSGGGGSGGYG QQDRGGR
NES-YK9-mAG-FUS(1–216)AllS	NES-YK9-mAG(same as the above sequence)-VDMASNDSTQQATQSSGASPTQPGQGSSQQSSQPSGQQSSSG SSQSTDTSGSGQSSSSSSGQSQNTGSGTQSTPQGSGSTGGSGSS QSSQSSSGQQSSSPGSGQQPAPSSTSGSSGSSSQSSSSGQPQSGS

	SSQQPSSGGQQQSSGQQQSSNPPQGSGQQNQSNSSSGGGGGGGGGGNSGQDQSSMSSGGGSGGGSGNQDQSGGGGSGGSGQQDRGGR
NES-YK9-mCh-Hdj1	MDLQKKLEELELDEQQKKRLGGSGGYKYKYKYKYGGSEFMVSKGEEDNMAIIKEFMRFKVHMEGSVNGHEFEIEGEGEGRPYEGTQTAKLKVTKGGPLPFAWDILSPQFMYGSKAYVKHPADIPDYLKLSFPEGFKWERVMNFEDGGVVTVTQDSSLQDGEFIYKVKLRGTNFPSDGPVMQKKTMGWEASSERMYPEDGALKGEIKQRLKLKDGGHYDAEVKTTYKAKKPVQLPGAYNVNIKLDITSHNEDYTIVEQYERAEGRHSTGGMDELYKVDMGKDYYQTLGLARGASDEEIKRAYRRQALRYHPDKNKEPGAEEKFKEIAEAYDVLSDPRKREIFDRYGEEGLKGSGPSGGSGGGANGTSFSYTFHGDPHAMFAEFFGGRNPFDTFFGQRNGEEGMDIDDPFSGFPMGMGGFTNVNFGRSRSAQEPARKKQDPPVTHDLRVSLEEIYSGCTKKMKISHKRLNPDGKSIRNEDKILTIEVKKGWKEGTKITFPKEGDQTSNNIPADIVFVLKDKPHNIFKRDGSDVIYPARISLREALCGCTVNVPTLDGRTIPVVFKDVIRPGMRRKVPGEGLPLPKTPEKRGDLIIEFEVIFPERIPQTSRTVLEQVLPI
NES-YK13-mAG-HOP	NES-YK13-mAG(same as the above sequence)-VEMEQVNELKEKGNKALSVGNIDDALQCYSEAIKLDPHNHVLYSNRSAAYAKKGDYQKAYEDGCKTVDLKPDWGKGYSRKAAALEFLNRFEEAKRTYEEGLKHEANNPQLKEGLQNMEARLAERKFMNPFNMPNLYQKLESDPRTRTLLSDPTYRELIEQLRNKPSDLGTKLQDPRIMTTLSVLLGVDLGSMDEEEEIATPPPPPPPKKETKPEPMEEDLPENKKQALKEKELGNDAYKKKDFDTALKHYDKAKELDPTNMTYITNQAAVYFEKGDYNKCRELCEKAIEVGRENREDYRQIAKAYARIGNSYFKEEKYKDAIHFYNKSLAEHRTPDVLKKCQQAEKILKEQERLAYINPDLALEEKNKGNECFQKGDYPQAMKHYTEAIKRNPKDAKLYSNRAACYTKLLEFQLALKDCEECIQLEPTFIKGYTRKAAALEAMKDYTKAMDVYQKALDLDSSCKEAADGYQRCMMAQYNRHDSPEDVKRRAMADPEVQQIMSDPAMRLILEQMQKDPQALSEHLKNPVIAQKIQKLMDVGLIAIR
NES-YK13(K6Y/Y7K)-mAG-HOP	MDLQKKLEELELDEQQKKRLGGSGGYKYKY<u>YK</u>KYKYKYGGSEF-mAG(same as the above sequence)-VEMEQVNELKEKGNKALSVGNIDDALQCYSEAIKLDPHNHV

	LYSNRSAAYAKKGDYQKAYEDGCKTVDLKPDWGKGYSRKA<br>AALEFLNRFEEAKRTYEEGLKHEANNPQLKEGLQNMEARLA<br>ERKFMNPFNMPNLYQKLESDPRTRTLLSDPTYRELIEQLRNKP<br>SDLGTKLQDPRIMTTLSVLLGVDLGSMDEEEEIATPPPPPPPKK<br>ETKPEPMEEDLPENKKQALKEKELGNDAYKKKDFDTALKHY<br>DKAKELDPTNMTYITNQAAVYFEKGDYNKCRELCEKAIEVG<br>RENREDYRQIAKAYARIGNSYFKEEKYKDAIHFYNKSLAEHRT<br>PDVLKKCQQAEKILKEQERLAYINPDLALEEKNKGNECFQKG<br>DYPQAMKHYTEAIKRNPKDAKLYSNRAACYTKLLEFQLALK<br>DCEECIQLEPTFIKGYTRKAAALEAMKDYTKAMDVYQKALD<br>LDSSCKEAADGYQRCMMAQYNRHDSPEDVKRRAMADPEVQ<br>QIMSDPAMRLILEQMQKDPQALSEHLKNPVIAQKIQLMDVG<br>LIAIR
NES-YK13-mAG-HOPΔTPR1(119-543)	NES-YK13-mAG(same as the above sequence)-<br>VEMLAERKFMNPFNMPNLYQKLESDPRTRTLLSDPTYRELIEQ<br>LRNKPSDLGTKLQDPRIMTTLSVLLGVDLGSMDEEEEIATPPP<br>PPPPKKETKPEPMEEDLPENKKQALKEKELGNDAYKKKDFDT<br>ALKHYDKAKELDPTNMTYITNQAAVYFEKGDYNKCRELCEK<br>AIEVGRENREDYRQIAKAYARIGNSYFKEEKYKDAIHFYNKSL<br>AEHRTPDVLKKCQQAEKILKEQERLAYINPDLALEEKNKGNE<br>CFQKGDYPQAMKHYTEAIKRNPKDAKLYSNRAACYTKLLEF<br>QLALKDCEECIQLEPTFIKGYTRKAAALEAMKDYTKAMDVY<br>QKALDLDSSCKEAADGYQRCMMAQYNRHDSPEDVKRRAM<br>ADPEVQQIMSDPAMRLILEQMQKDPQALSEHLKNPVIAQKIQ<br>KLMDVGLIAIR
NES-YK13-mAG-HOPΔTPR1-DP1(201-543)	NES-YK13-mAG(same as the above sequence)-<br>VEMPPPPPKKETKPEPMEEDLPENKKQALKEKELGNDAYKKK<br>DFDTALKHYDKAKELDPTNMTYITNQAAVYFEKGDYNKCRE<br>LCEKAIEVGRENREDYRQIAKAYARIGNSYFKEEKYKDAIHFY<br>NKSLAEHRTPDVLKKCQQAEKILKEQERLAYINPDLALEEKN<br>KGNECFQKGDYPQAMKHYTEAIKRNPKDAKLYSNRAACYTK<br>LLEFQLALKDCEECIQLEPTFIKGYTRKAAALEAMKDYTKAM<br>DVYQKALDLDSSCKEAADGYQRCMMAQYNRHDSPEDVKRR<br>AMADPEVQQIMSDPAMRLILEQMQKDPQALSEHLKNPVIAQK<br>IQKLMDVGLIAIR

NES-YK13-mAG-HOPΔTPR2B-DP2(1-356)	NES-YK13-mAG(same as the above sequence)-<br>VEMEQVNELKEKGNKALSVGNIDDALQCYSEAIKLDPHNHV<br>LYSNRSAAYAKKGDYQKAYEDGCKTVDLKPDWGKGYSRKA<br>AALEFLNRFEEAKRTYEEGLKHEANNPQLKEGLQNMEARLA<br>ERKFMNPFNMPNLYQKLESDPRTRTLLSDPTYRELIEQLRNKP<br>SDLGTKLQDPRIMTTLSVLLGVDLGSMDEEEEIATPPPPPPPKK<br>ETKPEPMEEDLPENKKQALKEKELGNDAYKKKDFDTALKHY<br>DKAKELDPTNMTYITNQAAVYFEKGDYNKCRELCEKAIEVG<br>RENREDYRQIAKAYARIGNSYFKEEKYKDAIHFYNKSLAEHRT<br>PDVLKKCQQAEKILKEQERLAYIN
NES-YK13-mAG-TPR1-DP1(1-200)	NES-YK13-mAG(same as the above sequence)-<br>VEMEQVNELKEKGNKALSVGNIDDALQCYSEAIKLDPHNHV<br>LYSNRSAAYAKKGDYQKAYEDGCKTVDLKPDWGKGYSRKA<br>AALEFLNRFEEAKRTYEEGLKHEANNPQLKEGLQNMEARLA<br>ERKFMNPFNMPNLYQKLESDPRTRTLLSDPTYRELIEQLRNKP<br>SDLGTKLQDPRIMTTLSVLLGVDLGSMDEEEEIATPP
NES-YK13-mAG-TPR1(1-118)	NES-YK13-mAG(same as the above sequence)-<br>VEMEQVNELKEKGNKALSVGNIDDALQCYSEAIKLDPHNHV<br>LYSNRSAAYAKKGDYQKAYEDGCKTVDLKPDWGKGYSRKA<br>AALEFLNRFEEAKRTYEEGLKHEANNPQLKEGLQNMEAR
NES-YK13-mAG-DP1(119-200)	NES-YK13-mAG(same as the above sequence)-<br>VEMLAERKFMNPFNMPNLYQKLESDPRTRTLLSDPTYRELIEQ<br>LRNKPSDLGTKLQDPRIMTTLSVLLGVDLGSMDEEEEIATPP
His-HOP	MGSSHHHHHHSSGLVPRGSHMASMEQVNELKEKGNKALSV<br>GNIDDALQCYSEAIKLDPHNHVLYSNRSAAYAKKGDYQKAYE<br>DGCKTVDLKPDWGKGYSRKAAALEFLNRFEEAKRTYEEGLK<br>HEANNPQLKEGLQNMEARLAERKFMNPFNMPNLYQKLESDP<br>RTRTLLSDPTYRELIEQLRNKPSDLGTKLQDPRIMTTLSVLLGV<br>DLGSMDEEEEIATPPPPPPPKKETKPEPMEEDLPENKKQALKE<br>KELGNDAYKKKDFDTALKHYDKAKELDPTNMTYITNQAAVY<br>FEKGDYNKCRELCEKAIEVGRENREDYRQIAKAYARIGNSYF<br>KEEKYKDAIHFYNKSLAEHRTPDVLKKCQQAEKILKEQERLA<br>YINPDLALEEKNKGNECFQKGDYPQAMKHYTEAIKRNPKDA<br>KLYSNRAACYTKLLEFQLALKDCEECIQLEPTFIKGYTRKAAA<br>LEAMKDYTKAMDVYQKALDLDSSCKEAADGYQRCMMAQY

NRHDSPEDVKRRAMADPEVQQIMSDPAMRLILEQMQKDPQA
LSEHLKNPVIAQKIQKLMDVGLIAIR

Table S9 Primer DNAs for cloning.

Protein	Fragment	Forward Sequence	Reverse Sequence
FUS(1-216)	1st PCR	AAAGCTGTTCCAGAACCTGG	CGCCACCACTGCCACCCCTGCCGC
	2nd PCR	GTTGTCGACATGGCCTCAAACGATTATAC	CAAGCGGCCGCTTAGCGGCCTCCACGGTCCTGCT
Hdj1	1st PCR	GGACGGCGACAGCGGGTCG	GGAAAGGTCCCTGGTCAGTCCTTGG
	2nd PCR	GAAGTCGACATGGGTAAAGACTACTACCAGACG	CTTGCGGCCGCCTATATTGGAAGAACCTGCTC
HOP(1-543)	For pET-vector	GTTGCTAGCATGGAGCAGGTCAATGAGC	CAAGCGGCCGCTCACCGAATTGCAATCAG
HOP(119-543)		GAACTCGAGATGTTGGCAGAGAGAAAATTCATG	CTTGCGGCCGCTCACCGAATTGCAATC
HOP(201-543)		GAACTCGAGATGCCACCACCCCCTC	CTTGCGGCCGCTCACCGAATTGCAATC
HOP(1-356)		GAACTCGAGATGGAGCAGGTCAATGAGCT	CTTGCGGCCGCTCAGTTTATGTAGGCC
HOP(1-200)		GAACTCGAGATGGAGCAGGTCAATGAGCT	CTTGCGGCCGCTCATGGAGGTGTTGCAATC
HOP(1-118)		GAACTCGAGATGGAGCAGGTCAATGAGCT	CTTGCGGCCGCTCACCTGGCCTCCATATTCTG
HOP(119-200)		GAACTCGAGATGTTGGCAGAGAGAAAATTCATG	CTTGCGGCCGCTCATGGAGGTGTTGCAATC

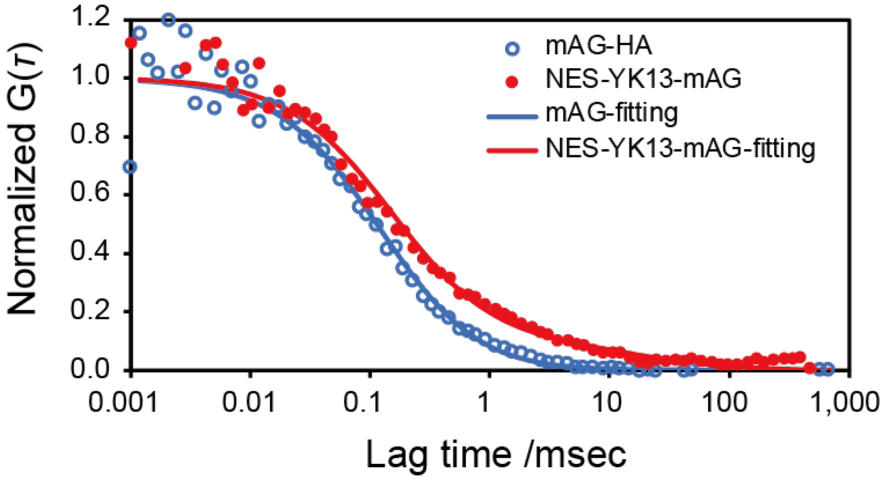

Figure S1. Live cell FCS analysis of mAG-HA and NES-YK13-mAG. The normalized fluorescence autocorrelation fractions of mAG-HA (blue open circles) and NES-YK13-mAG (red circles) are plotted. FCS measurements were performed on the Leica TCS SP8 confocal microscope system (LAS X 3.5.5. 19976 system) with an HC PL APO CS2 63.0×/1.2 water-immersion objective. The confocal pinhole diameter was adjusted to 1 airy unit. The mAG-HA or NES-YK13-mAG was excited by a 488-nm Argon laser. The laser power was tuned to adjust the count rate to 100-200 kHz. For each position, we set a measuring time of 10 seconds. Data were analyzed using SymPhoTime® (Version 5.3.2.2) software. The acquired auto-correlation curves of mAG-HA or NES-YK13-mAG were fitted with a one-component or two-component model in which the τ_{short} was set as 155 μsec, corresponding to the diffusion time of mAG-HA.

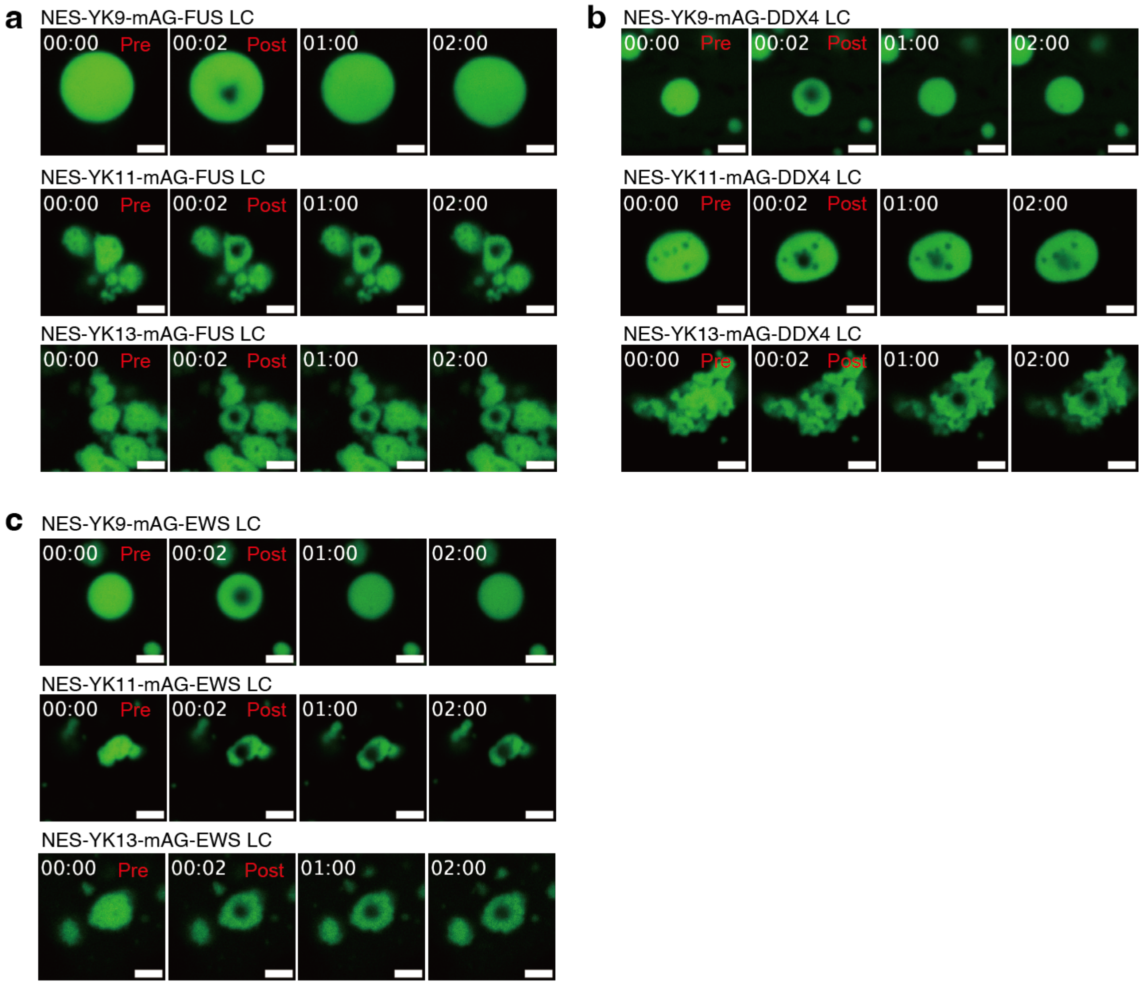

Figure S2. FRAP analysis of NES-YK-mAG-LCs in COS-7 cells. The droplets of (a) FUS LC, (b) DDX4 LC, or (c) EWS LC-fused NES-YK-mAG in COS-7 cells were tested by FRAP analysis. The scale bars mean 2 μm.

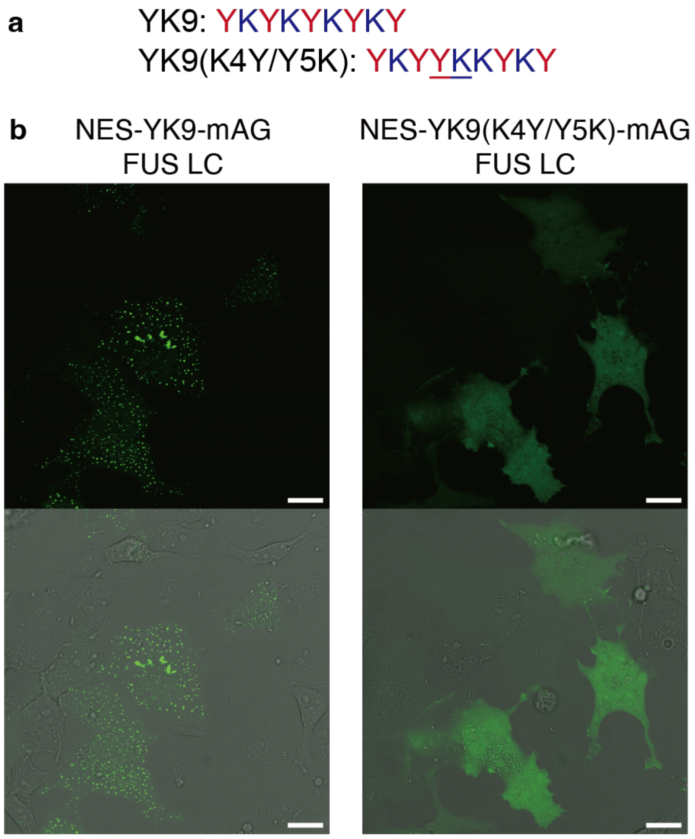

Figure S3. Fluorescence images of COS-7 cells expressing NES-YK9(K4Y/Y5K)-mAG FUS LC. The scale bars mean 20 μm.

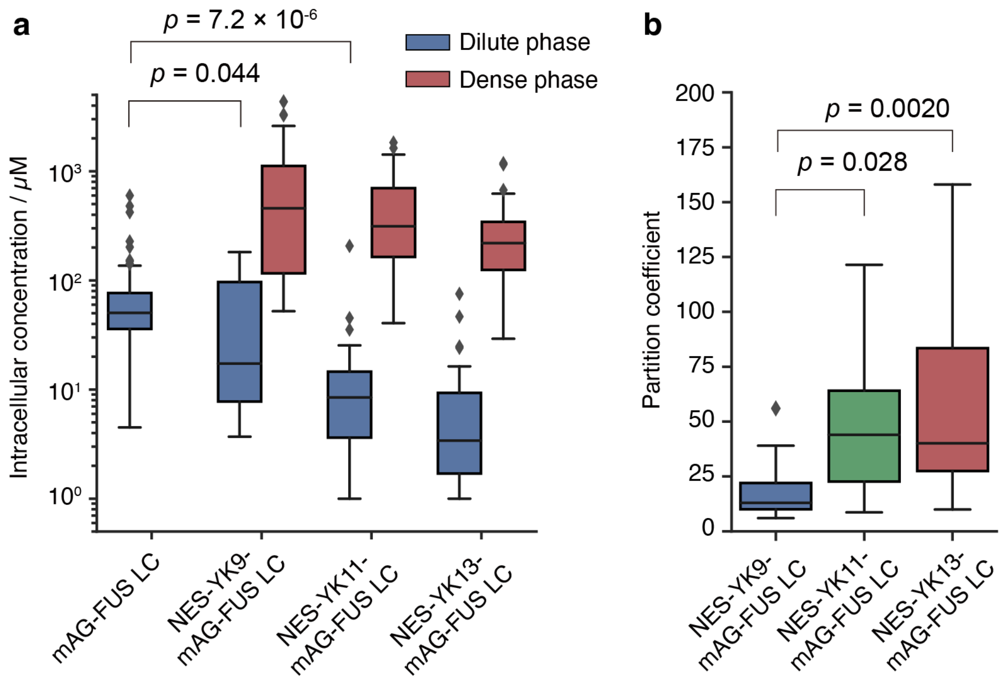

Figure S4. Quantification of intracellular mAG-FUS LC concentrations and partition coefficient in COS-7 cells. (a) Intracellular concentrations of mAG-FUS LC (without YK tag) and NES-YK9-, YK11-, and YK13-mAG-FUS LC in COS-7 cells. Protein concentrations were separately quantified in the dilute and dense phases. (b) Partition coefficients of NES-YK-tagged mAG FUS LC constructs, calculated as the ratio of fluorescence intensity inside versus outside the droplets. Sample sizes were as follows: n = 49 (mAG-FUS LC), 56 (YK9 fusion), 46 (YK11 fusion), and 60 cells (YK13 fusion). The box plot is represented with the center line, median; box limits, Q1 and Q3; whiskers, 1.5×interquartile range; points, outliers. Statistical comparisons between the two groups were performed using unpaired two-tailed Student's t-tests.

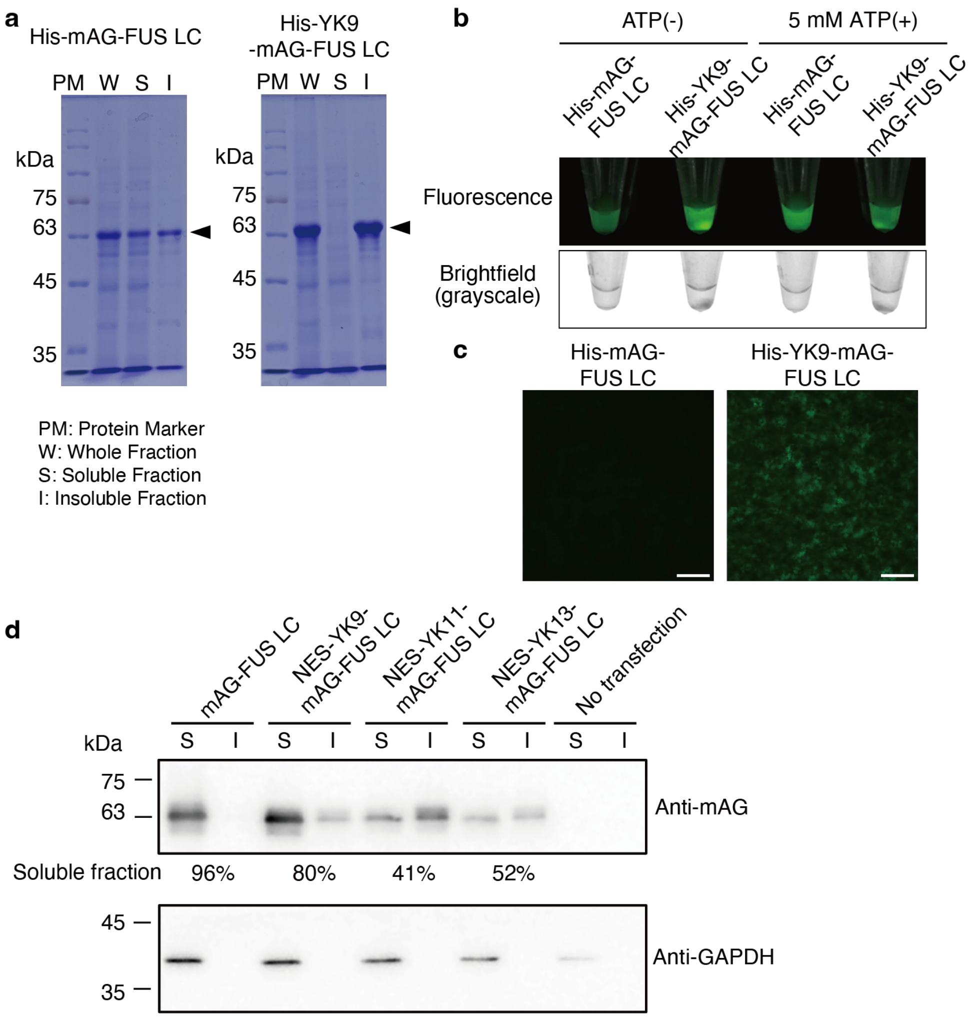

Figure S5. YK-fused FUS LC forms aggregates in vitro but remains soluble in mammalian cells. (a) SDS-PAGE analysis of His-mAG-FUS LC and His-YK9-mAG-FUS LC expressed in *E. coli*. Whole cell lysate (W) was separated into soluble fraction (S), and insoluble pellet (I). The arrowheads indicate the target proteins. (b) Test tube observation of in vitro-assembled His-mAG-FUS LC and His-YK9-mAG-FUS LC in the absence or presence of ATP. Brightfield and fluorescence images demonstrate that YK fusion induces the aggregation of FUS LC in vitro. (c) Fluorescence microscopy of in vitro protein solutions confirms that His-YK9-mAG-FUS LC forms visible aggregates, while His-mAG-FUS LC remains dispersed. The scale bars mean 200 μm. (d) Western blot analysis of mAG-FUS LC and YK-tagged variants in soluble (S) and insoluble (I) fractions. Percentages indicate the proportion of protein in the soluble fraction. GAPDH was used as a loading control.

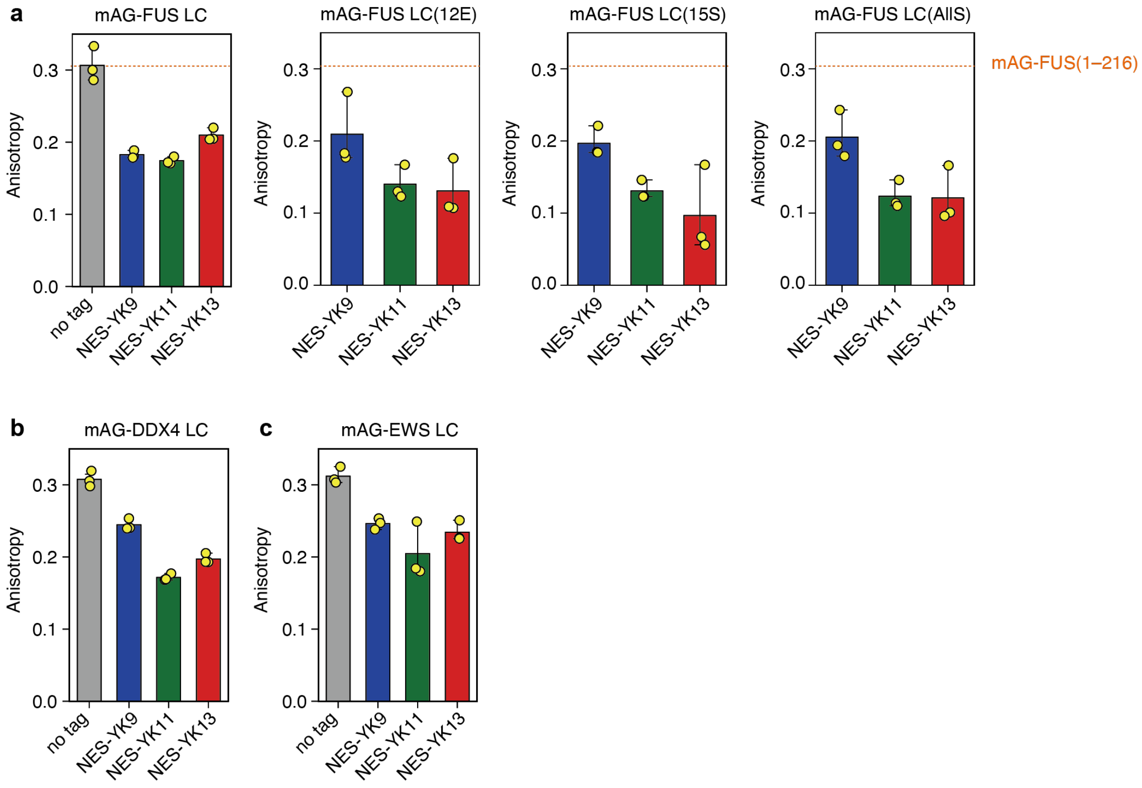

Figure S6. Fluorescence anisotropy of NES-YK-mAGs-LCs in COS-7 cells. Dependence of fluorescence anisotropy on YK peptide length (a) FUS LC, FUS LC(12E), FUS LC(15S), and FUS LC(AllS) mutants, (b) DDX4 LC, and (c) EWS LC. The dashed line indicates the anisotropy level of mAG-FUS LC without any tag. Data are presented as mean values +/- SD (*n* = 3, independent samples).

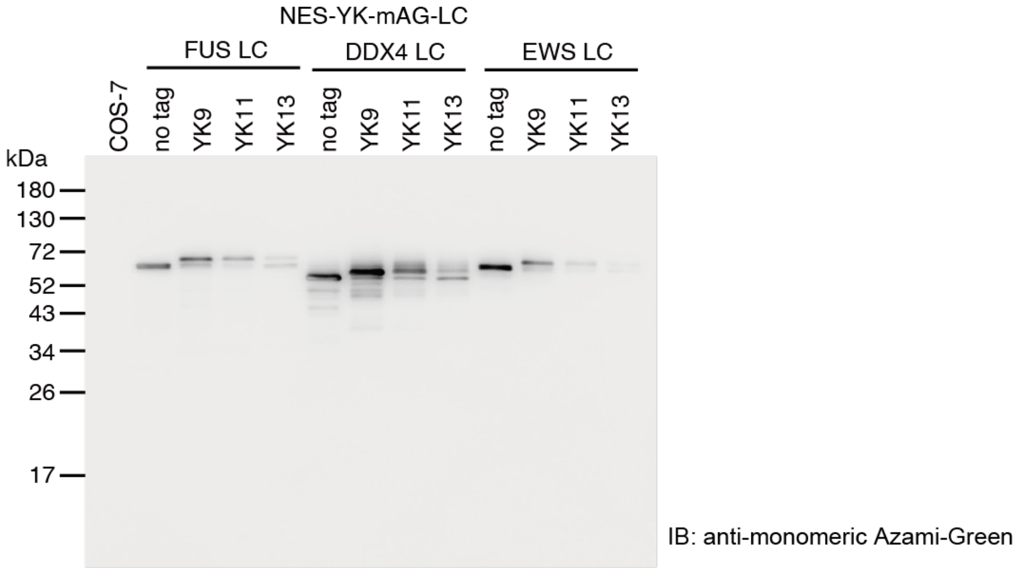

Figure S7. Western blotting of COS-7 cells expressing NES-YK-mAG-LC. Two days after transfection, the cells were lysed in SDS-PAGE sample buffer (TCI, B5834) with 100 mM DTT. The samples were applied to SDS-PAGE (12.5% AA) and transferred onto a PVDF membrane (Millipore). The membrane was soaked in 5% skimmed milk containing PBS-T buffer for 30 min at room temperature, then incubated with anti-monomeric Azami-Green 1 pAb (PM052M, MBL, 1:2000 dilution) at 4 ºC overnight. After washing with PBS-T buffer three times, we incubated with goat anti-mouse IgG-HPR conjugate (Promega, W402B, 1:2,500 dilution) in PBS-T. After three times washing, we soaked it in the ECL prime (RPN2232) working solution. The chemiluminescence was detected by LuminoGraph II EM instrument (ATTO) with ImageSaver7.

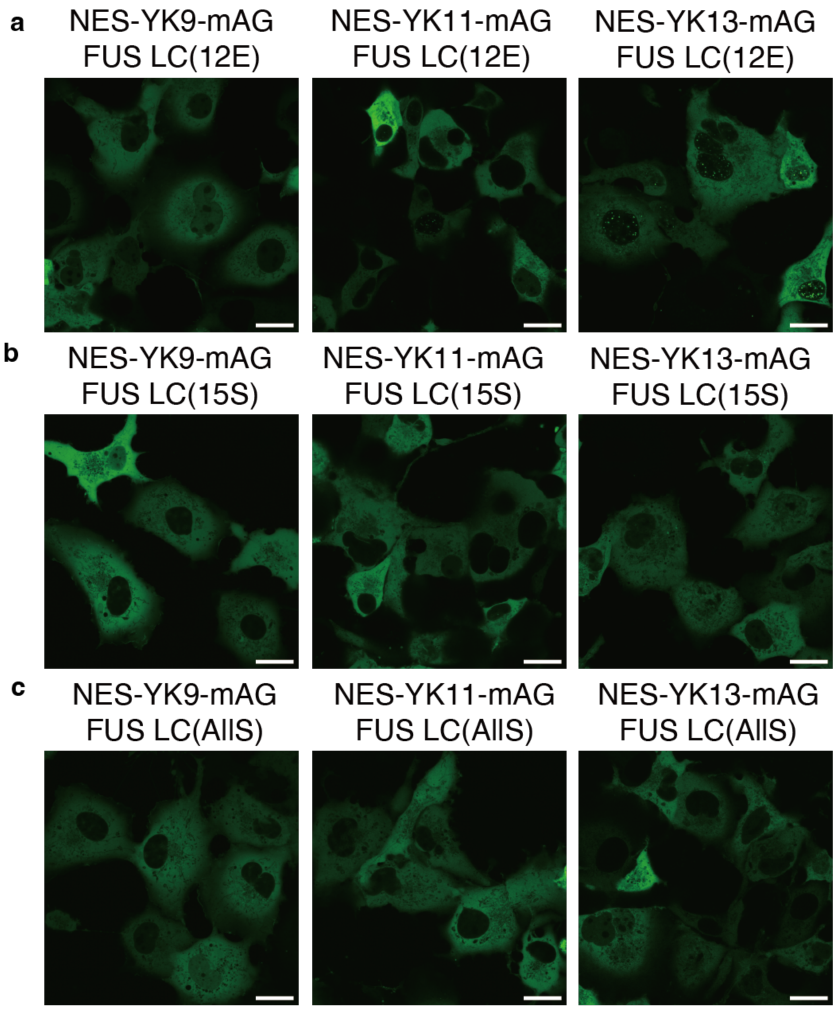

Figure S8. Fluorescence images of COS-7 cells expressing NES-YK-mAG FUS LC mutants. The FUS LC(12E), FUS LC(15S), or FUS LC(AllS)-fused NES-YK-mAG in COS-7 cells were observed by CLSM. The scale bars mean 20 μm.

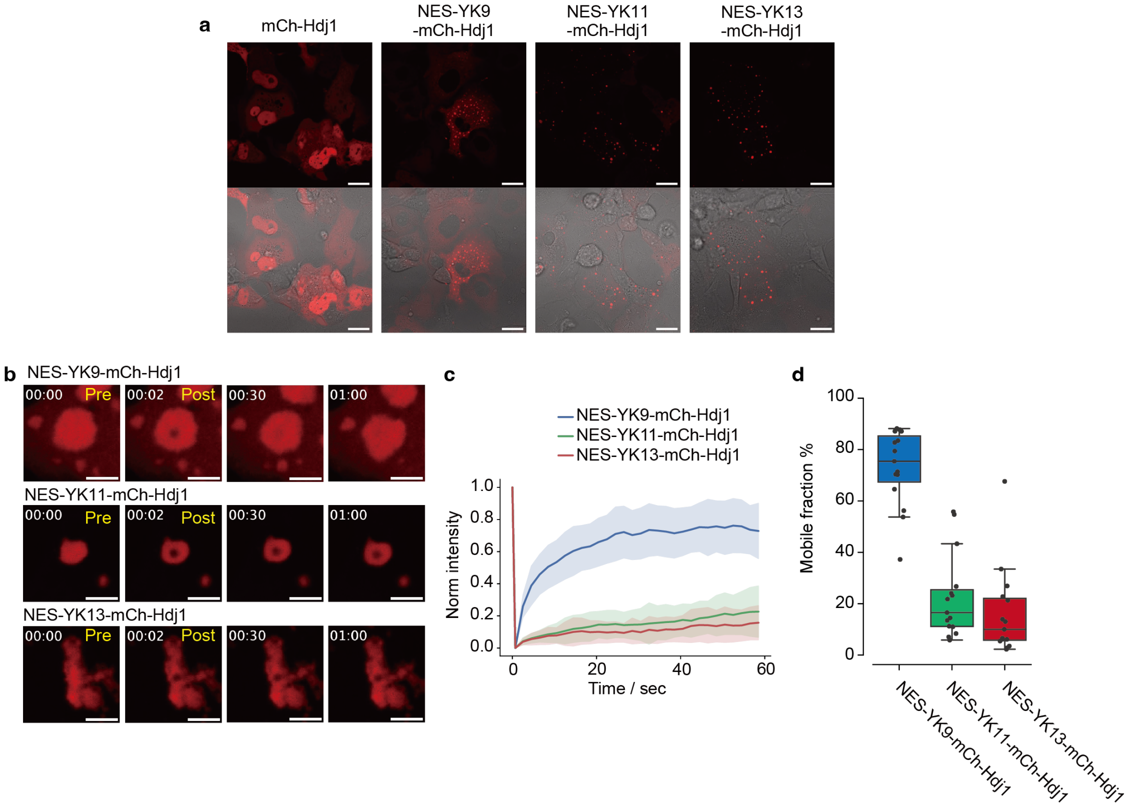

Figure S9. Droplet formation of YK-tagged Hdj1 in living cells. a, Fluorescence images of NES-YK-mCh fused Hdj1 in COS-7 cells. The scale bars mean 20 µm. **b,** FRAP analysis of droplets of NES-YK-mCh fused Hdj1. The scale bars mean 3 µm. **c,** Time course of normalized fluorescence intensities of bleached regions. The data points are presented as mean values with +/- S.D. as translucent error bands (*n* = 15 cells). **d,** Mobile fractions obtained from FRAP analysis. The box plots are presented as follows: central line, median; box limits, Q1 and Q3; whiskers, 1.5×interquartile range; and points, outliers. (*n* = 15 cells).

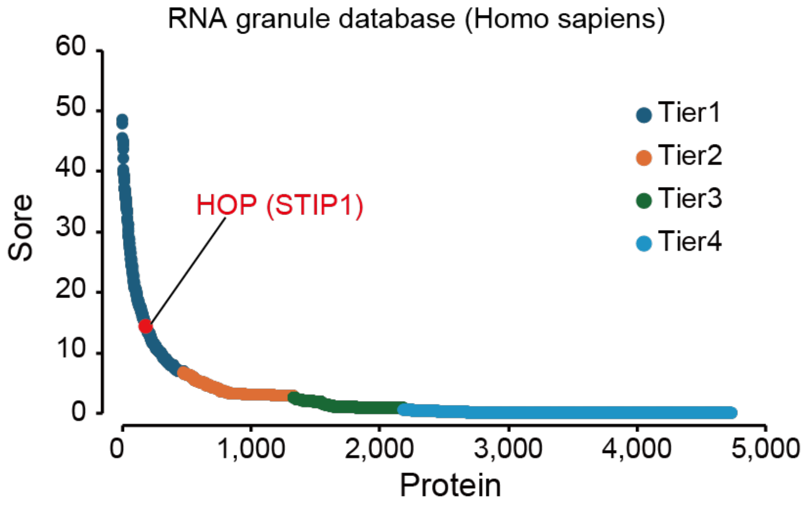

Figure S10. HOP (STIP1) in RNA granule database. The human genes in the database were sorted by the score. STIP1 (HOP) is categorized as Tier 1. The STIP1 (HOP) score is 14.5, which is 184th in 4731 genes.

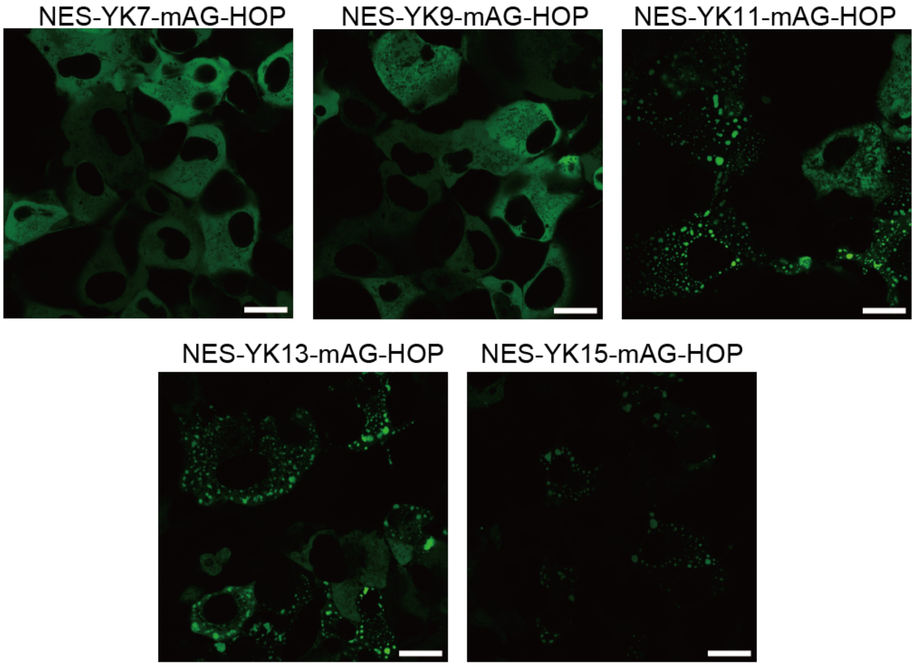

Figure S11. Fluorescence images of COS-7 cells expressing NES-YK-mAG-HOPs. Two days after transfection, COS-7 cells were observed by using CLSM. The scale bars mean 20 µm.

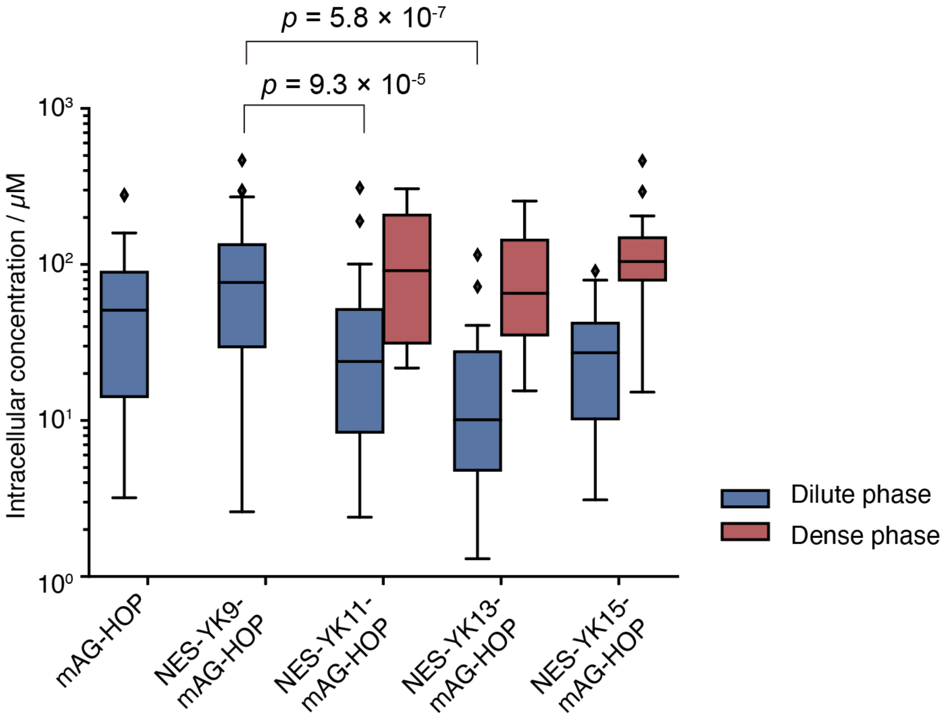

Figure S12. Quantification of intracellular mAG-HOP concentrations in COS-7 cells. (a) Intracellular concentrations of mAG-HOP (without YK tag) and NES-YK9-, YK11-, YK13-, and YK15-mAG-HOP in COS-7 cells. Protein concentrations were separately quantified in the dilute and dense phases. Sample sizes were as follows: n = 56 (mAG-HOP), 45 (YK9 fusion), 53 (YK11 fusion, dilute phase), 30 (YK11 fusion, dense phase), 51 (YK13 fusion, dilute phase), 36 (YK13 fusion, dense phase), 47 (YK15 fusion, dilute phase), and 27 (YK15 fusion, dense phase). The box plot is represented with the center line, median; box limits, Q1 and Q3; whiskers, 1.5×interquartile range; points, outliers. Statistical comparisons between the two groups were performed using unpaired two-tailed Student's t-tests.

a YK13: YKYKYKYKYKYKY

YK13(K6Y/Y7K): YKYKYYKKYKYKY

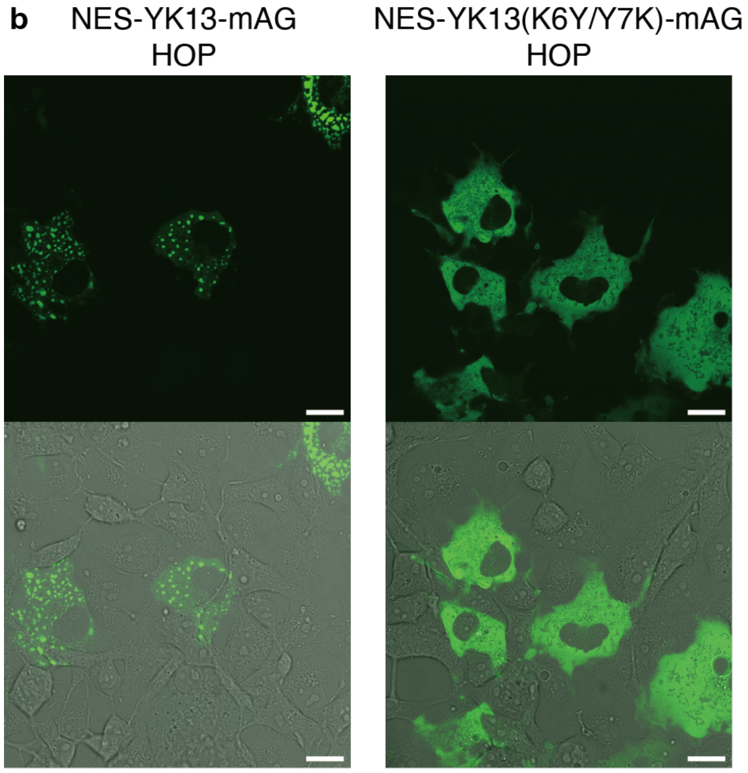

Figure S13. Fluorescence images of COS-7 cells expressing NES-YK13(K6Y/Y7K)-mAG HOP. The scale bars mean 20 μm.

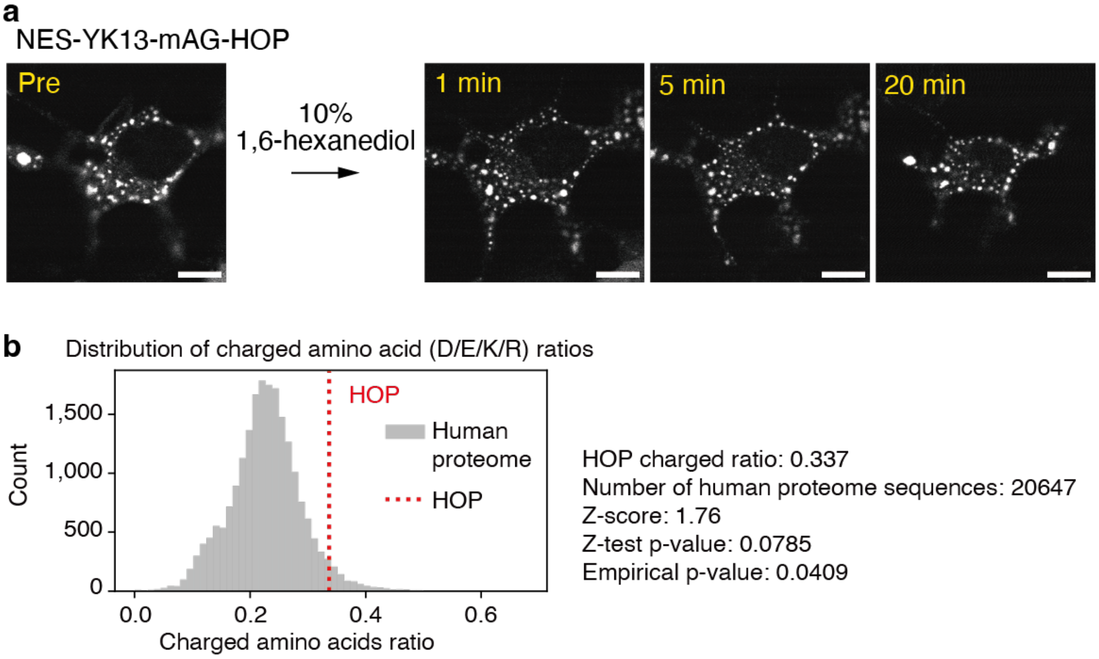

Figure S14. Resistance of NES-YK13-mAG-HOP droplets to 1,6-hexanediol treatment. (a) COS-7 cells expressing NES-YK13-mAG-HOP were imaged before and after treatment with 10% 1,6-hexanediol. Time-lapse fluorescence microscopy showed that the intracellular droplets persisted even 20 minutes post-treatment, indicating resistance to dissolution. The scale bars mean 10 µm. (b) Histogram showing the distribution of charged amino acid ratios (D, E, K, R) across the human proteome. The red dashed line marks the charged amino acid ratio for HOP (0.337), which is higher than average. This suggests that electrostatic interactions, rather than hydrophobic interactions, may play a major role in HOP-mediated droplet formation. Statistical analysis: Z-score = 1.76; Z-test p = 0.0785; Empirical p = 0.0409.

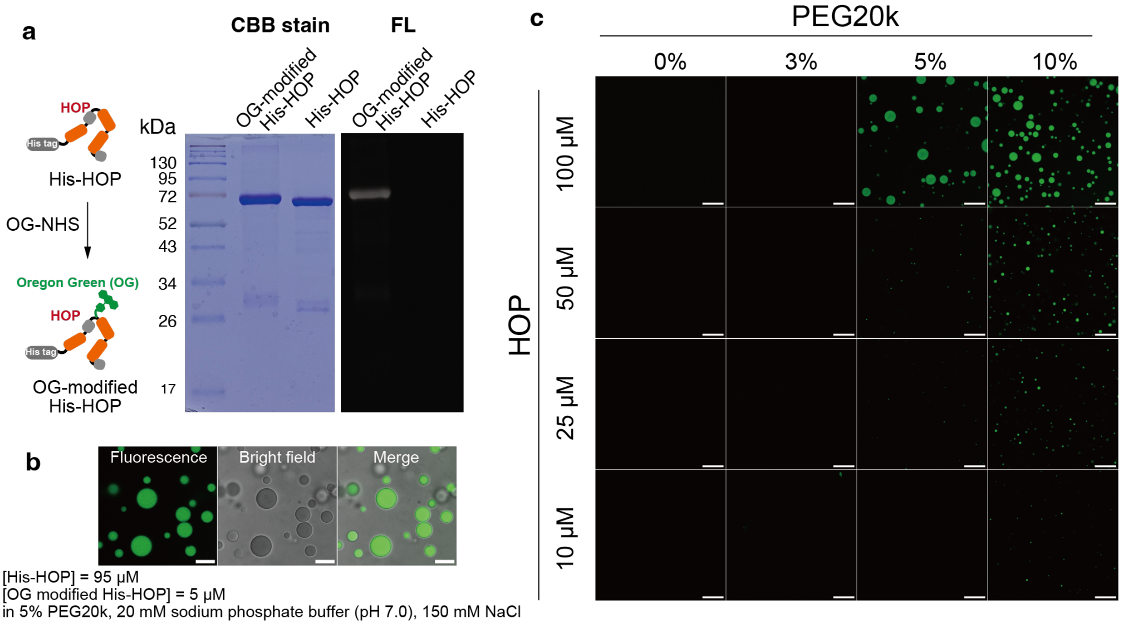

Figure S15. Droplet formation of His-HOP in test tubes. a, Oregon Green dye modification of His-HOP. SDS-PAGE (middle) and fluorescence gel images (right) of Oregon Green-modified His-HOP proteins and unmodified His-HOP. **b,** Droplet formation of His-HOP in the presence of PEG20k. The scale bars mean 10 μm. **c,** Dependence of HOP droplet formation on the HOP protein and PEG20k concentrations. Mixtures of His-HOP and Oregon Green-modified His-HOP (95:5 molar ratio) were diluted in various concentrations of PEG20k solution prepared in 20 mM sodium phosphate buffer with 150 mM NaCl (pH 7.0) in a 96-well plate. The scale bars mean 30 μm.

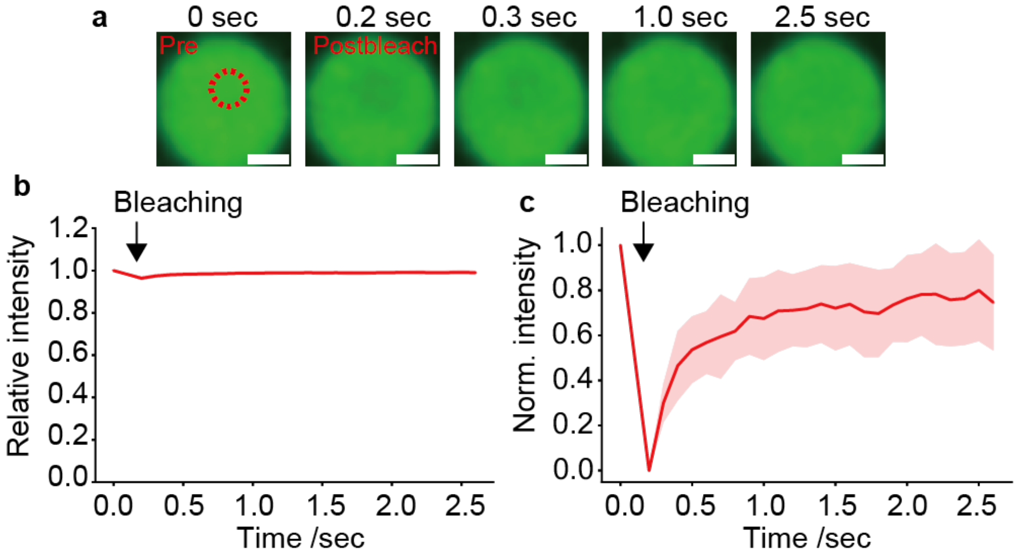

Figure S16. FRAP of His-HOP in test tubes. a, FRAP images of Oregon Green-modified His-HOP/His-HOP droplets. The scale bars mean 2 µm. **b,** Time course of relative fluorescence intensity of bleached regions. The fluorescence intensities at each time point were divided by the initial fluorescence value. **c,** Time course of normalized fluorescence intensities of bleached regions. The data points are presented as mean values with +/- S.D. as translucent error bands (*n* = 16 droplets).

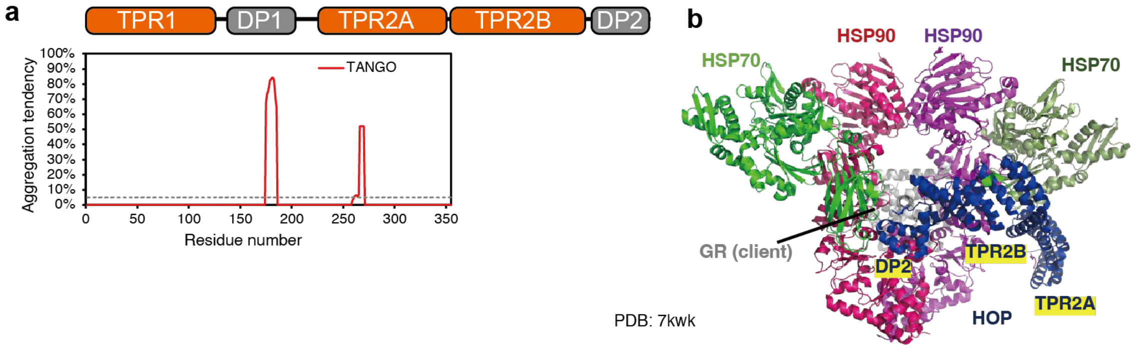

Figure S17. Prediction of aggregation tendency and structure of HOP. a, TANGO analysis of the HOP protein. Regions with more than five consecutive residues showing an aggregation tendency above 5% (dashed line) are defined as potential aggregation-prone regions. A strong peak is observed within the DP1 domain, suggesting a possible aggregation hotspot. **b**, Crystal structure of the Hsp90–Hsp70–HOP–GR (glucocorticoid receptor) complex (PDB: 7kwk). Although the full-length HOP was used for crystallization, only the C-terminal domains (TPR2A, TPR2B, DP2) were resolved in the structure.

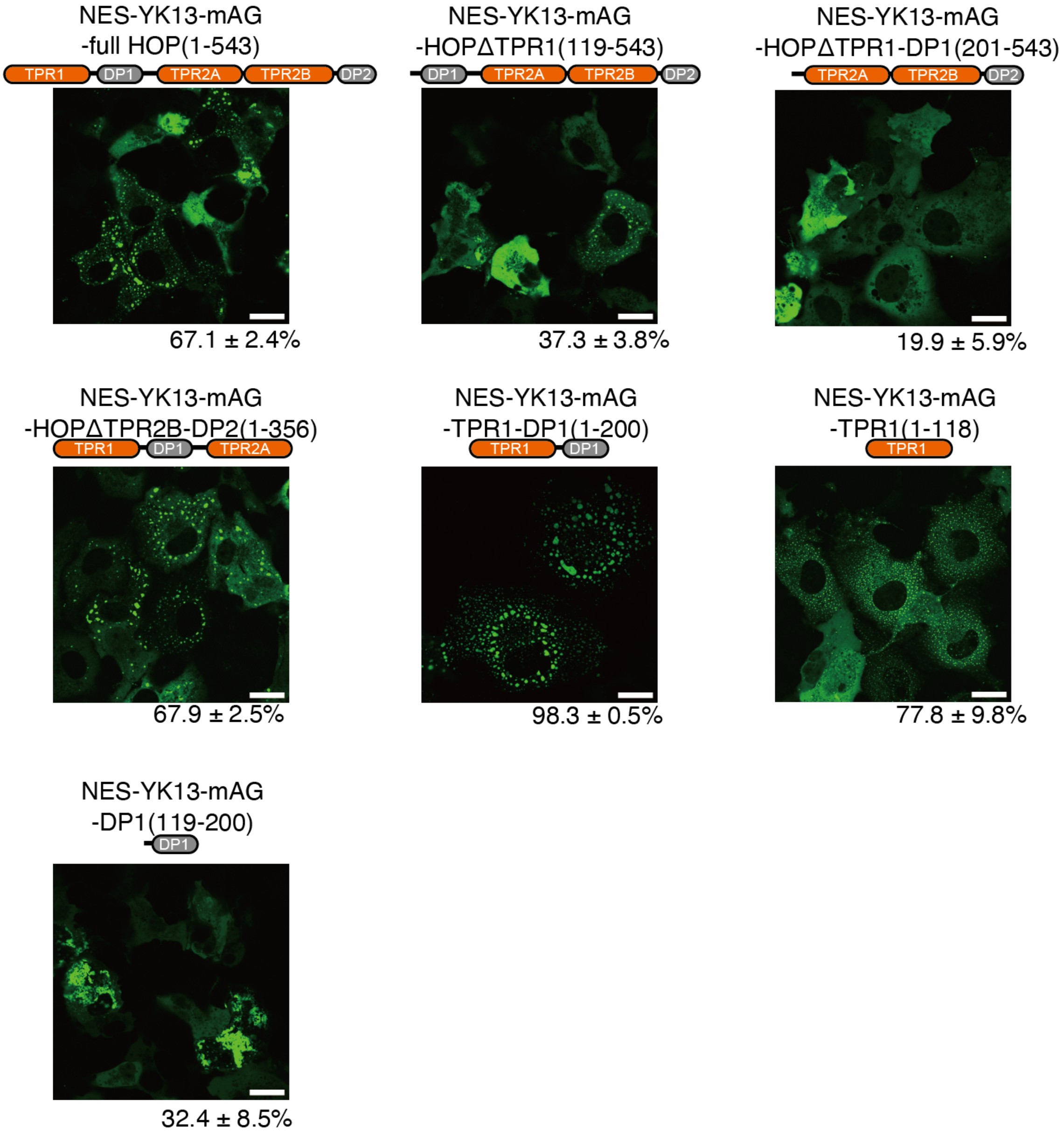

Figure S18. Fluorescence images of COS-7 cells expressing NES-YK13-mAG-fused truncated HOPs. The frequencies of cells showing fluorescent puncta in the total transfected cells were indicated below in each picture. The scale bars mean 20 µm.

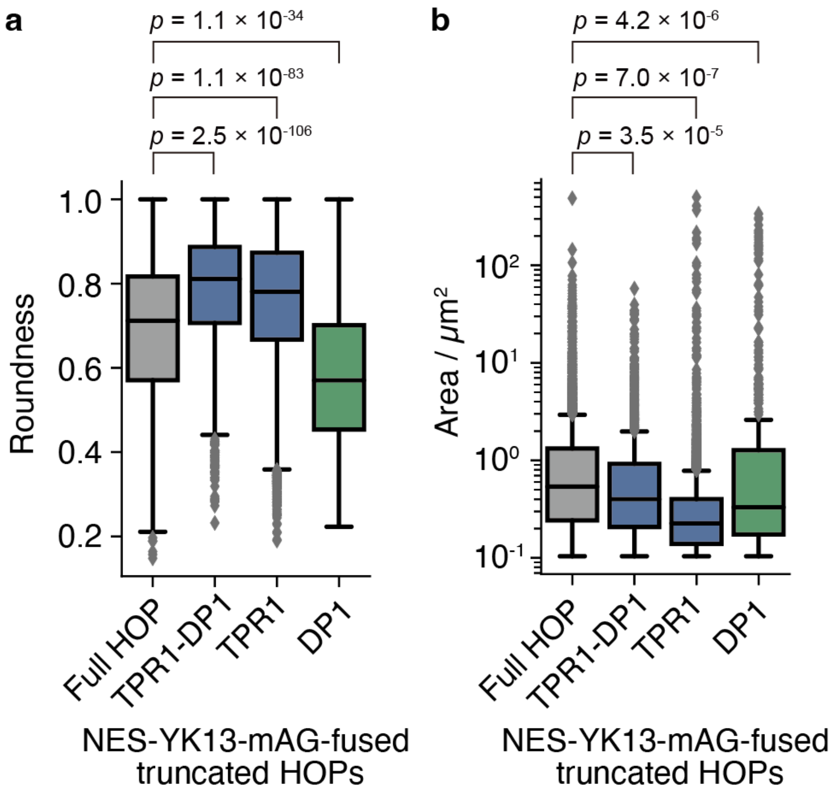

Figure S19. Roundness and size analysis of fluorescent puncta formed by HOP and truncated HOP fusions in living cells. (a) Roundness of fluorescent puncta. (b) Area of fluorescent puncta. Sample sizes were as follows: n = 3,270 (Full HOP), 2,026 (TPR1-DP1), 6,667 (TPR1), and 512 puncta (DP1), measured from nine or ten images per condition. The box plot is represented with the center line, median; box limits, Q1 and Q3; whiskers, 1.5×interquartile range; points, outliers. Statistical comparisons between the two groups were performed using unpaired two-tailed Student's t-tests.

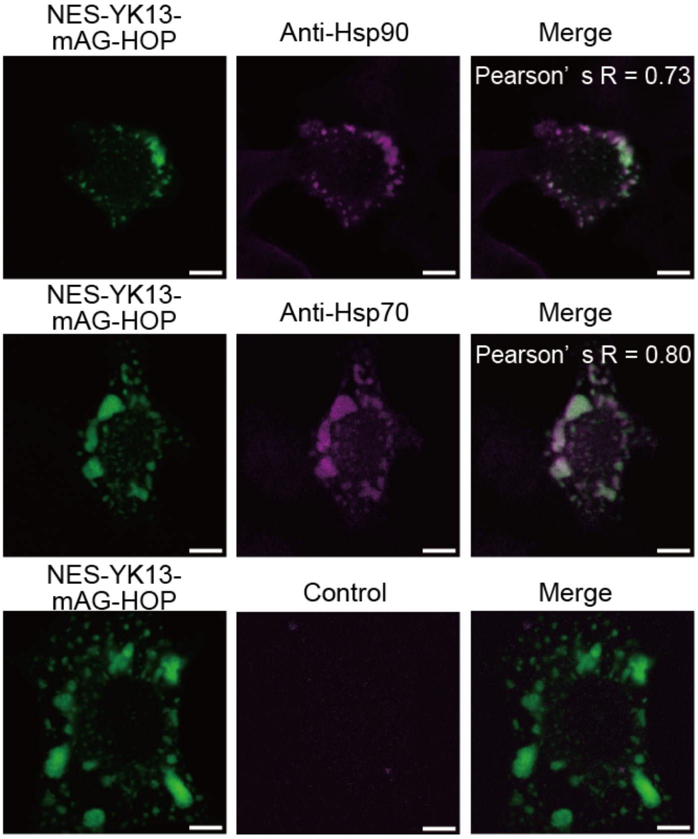

Figure S20. Colocalization of endogenous Hsp70 and Hsp90 with NES-YK13-mAG-HOP droplets. The Pearson’s R values for colocalization are shown in the merged images. The control experiment (bottom panel) shows that the colocalization of Hsp90 and Hsp70 with HOP-droplets is not attributed to fluorescent crosstalk between the two channels. The scale bars mean 5 µm.

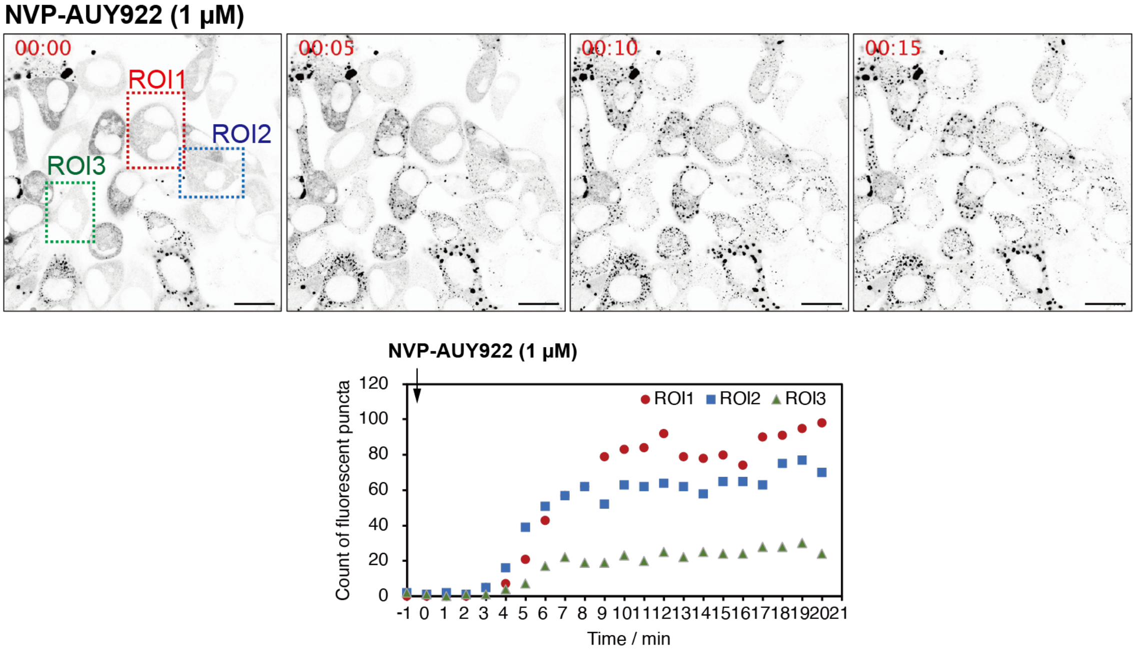

Figure S21. Hsp90 inhibitor NVP-AUY922 inducing NES-YK13-mAG-HOP aggregation. Time-lapse images of HeLa cells expressing NES-YK13-mAG-HOP before and after NVP-AUY922 (1 μM) treatments. The fluorescence is shown as inverted black-and-white images. The scale bars mean 20 μm. To count intracellular fluorescent puncta, the following image processing was performed using Fiji: 1) background subtraction (rolling ball radius 50.0 pixels), 2) threshold by ostu-method using the last image at 20 minutes of incubation, 3) watershed, and 4) analysis particles (size: 0.1 - infinity μm^2).

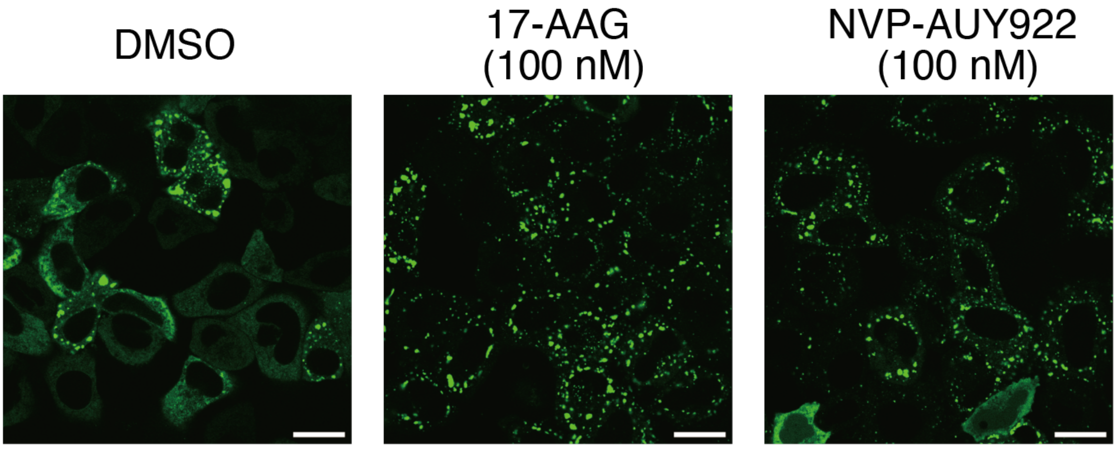

Figure S22. Increase in the frequency of cells exhibiting fluorescent puncta after Hsp90 inhibitor treatments. Fluorescence images of HeLa cells expressing NES-YK13-mAG-HOP after Hsp90 inhibitor addition. The scale bars mean 20 μm.

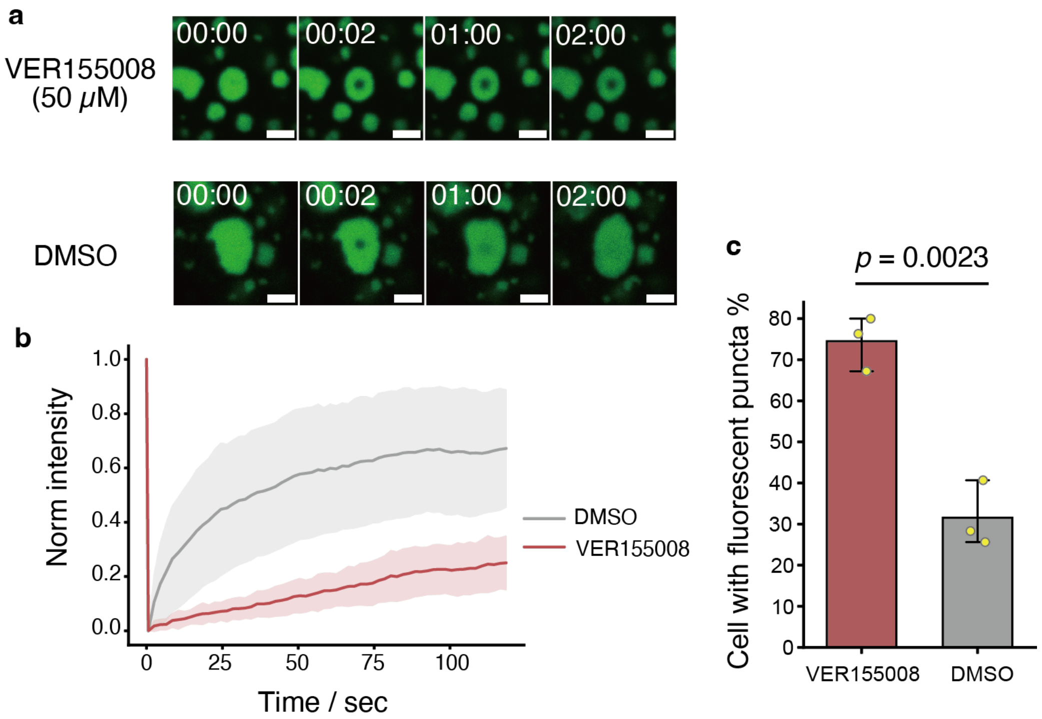

Figure S23. FRAP of NES-YK13-mAG-HOP droplets after Hsp70 inhibitor addition. a, Time-lapse images of FRAP. The scale bars mean 2 µm. **b,** Time course of normalized fluorescence intensities of bleached regions. The data points are presented as mean values with +/- S.D. as translucent error bands (*n* = 15 cells). **c,** Frequency of cells with fluorescent puncta. Treatment with VER155008 (Hsp70 inhibitor) increased the frequency of cells exhibiting fluorescent puncta (*n* = 3, biologically independent experiments). Data are presented as mean values +/− S.D. Statistical comparisons between the two groups were performed using unpaired two-tailed Student's t-tests.

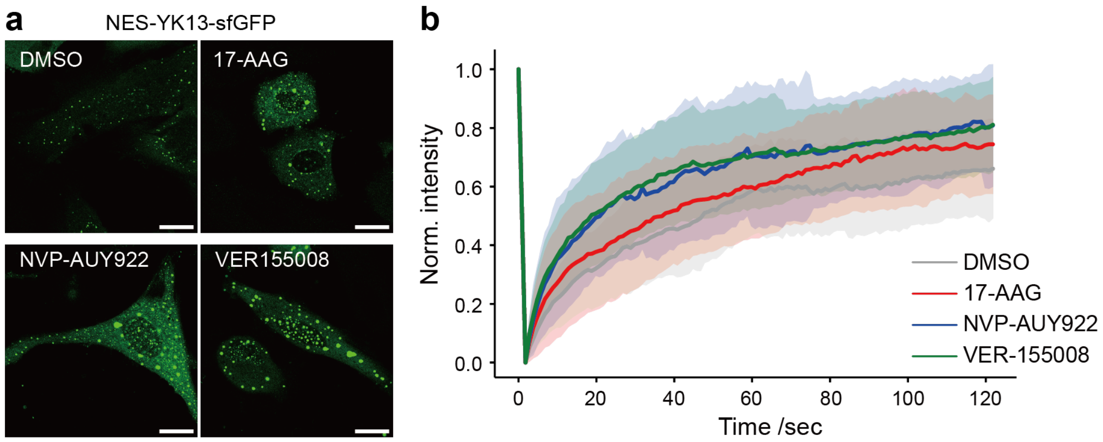

Figure S24. Hsp70 or Hsp90 inhibitors have no significant effect on NES-YK13-sfGFP droplets. **a,** Fluorescence images of HeLa cells expressing NES-YK13-sfGFP under different treatment conditions: DMSO, 17-AAG (100 nM), NVP-AUP922 (100 nM), and VER155008 (50 μM) for 30 min. The scale bars mean 20 μm. **b,** Time course of normalized fluorescence intensities of bleached regions. The data points are presented as mean values with +/- S.D. as translucent error bands (*n* = 15 cells).

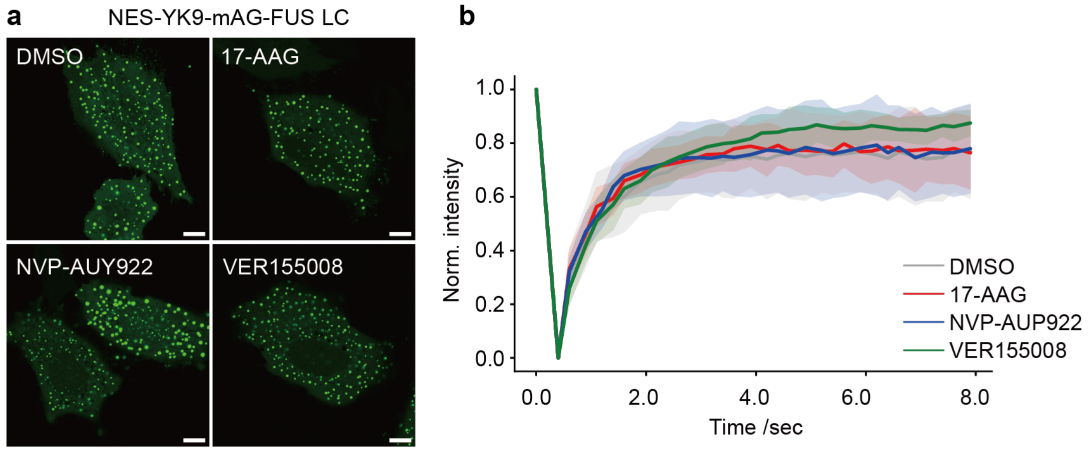

Figure S25. Hsp70 or Hsp90 inhibitors have no significant effect on NES-YK9-mAG-FUS LC droplets. a, Fluorescence images of HeLa cells expressing NES-YK9-mAG-FUS LC under different treatment conditions: DMSO, 17-AAG (100 nM), NVP-AUP922(100 nM), and VER155008 (50 µM) for 30 min. The scale bars mean 10 µm. **b,** Time course of normalized fluorescence intensities of bleached regions. The data points are presented as mean values with +/- S.D. as translucent error bands (*n* = 9 cells).

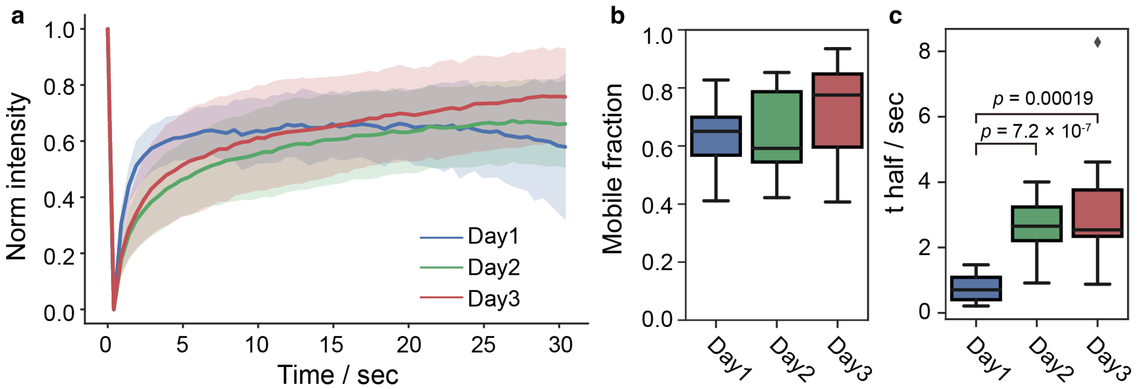

Figure S26. FRAP results of NES-YK9-mAG-FUS LC droplet on different incubation days. (a) Time course of normalized fluorescence intensities of bleached regions. The line plots represent the mean values with shaded error bands (S.D., *n* = 15 cells). (b) Mobile fraction obtained from FRAP analysis. (c) t half values. The box plot is represented with the center line, median; box limits, Q1 and Q3; whiskers, 1.5×interquartile range; points, outliers. Statistical comparisons between the two groups were performed using unpaired two-tailed Student's t-tests.

Reference

(1) Yang, Q.; Miki, T. *Characterization of Peptide-Fused Protein Assemblies in Living Cells*; Elsevier, 2024; Vol. 697. https://doi.org/10.1016/bs.mie.2024.01.022.

(2) Miki, T.; Hashimoto, M.; Nakai, T.; Mihara, H. A Guide-Tag System Controlling Client Enrichment into Y15 Peptide-Based Granules for an in-Cell Protein Recruitment Assay. *Chem. Commun.* **2021**, *57*, 11338–11341. https://doi.org/10.1039/d1cc03450b.

(3) Miki, T.; Hashimoto, M.; Takahashi, H.; Shimizu, M.; Nakayama, S.; Furuta, T.; Mihara, H. De Novo Designed Short Peptide Tags for Synthetic Protein Condensates in Mammalian Cells. *Nat. Commun.* **2024**, *15*, 8503. https://doi.org/10.1038/s41467-024-52708-5.
